# Myogenesis of Malacostraca – the “egg-nauplius” concept revisited

**DOI:** 10.1186/1742-9994-10-76

**Published:** 2013-12-11

**Authors:** Günther Joseph Jirikowski, Stefan Richter, Carsten Wolff

**Affiliations:** 1 Universität Rostock, Allgemeine & Spezielle Zoologie, Institut für Biowissenschaften, Universitaetsplatz 2, Rostock 18055, Germany; 2 AG Vergleichende Zoologie, Institut für Biologie, Humboldt-Universität zu Berlin, Philippstraße 13 Haus 2, Berlin 10115, Germany

## Abstract

**Background:**

Malacostracan evolutionary history has seen multiple transformations of ontogenetic mode. For example direct development in connection with extensive brood care and development involving planktotrophic nauplius larvae, as well as intermediate forms are found throughout this taxon. This makes the Malacostraca a promising group for study of evolutionary morphological diversification and the role of heterochrony therein. One candidate heterochronic phenomenon is represented by the concept of the ‘egg-nauplius’, in which the nauplius larva, considered plesiomorphic to all Crustacea, is recapitulated as an embryonic stage.

**Results:**

Here we present a comparative investigation of embryonic muscle differentiation in four representatives of Malacostraca: *Gonodactylaceus falcatus* (Stomatopoda), *Neocaridina heteropoda* (Decapoda), *Neomysis integer* (Mysida) and *Parhyale hawaiensis* (Amphipoda). We describe the patterns of muscle precursors in different embryonic stages to reconstruct the sequence of muscle development, until hatching of the larva or juvenile. Comparison of the developmental sequences between species reveals extensive heterochronic and heteromorphic variation. Clear anticipation of muscle differentiation in the nauplius segments, but also early formation of longitudinal trunk musculature independently of the teloblastic proliferation zone, are found to be characteristic to stomatopods and decapods, all of which share an egg-nauplius stage.

**Conclusions:**

Our study provides a strong indication that the concept of nauplius recapitulation in Malacostraca is incomplete, because sequences of muscle tissue differentiation deviate from the chronological patterns observed in the ectoderm, on which the egg-nauplius is based. However, comparison of myogenic sequences between taxa supports the hypothesis of a zoea-like larva that was present in the last common ancestor of Eumalacostraca (Malacostraca without Leptostraca). We argue that much of the developmental sequences of larva muscle patterning were retained in the eumalacostracan lineage despite the reduction of free swimming nauplius larvae, but was severely reduced in the peracaridean clade.

## Introduction

Malacostraca comprises approximately 30.000 species with a broad range of morphological and ecological diversity. Throughout the malacostracan clade an enormous variety of reproductive strategies can be found. Malacostracan ontogeny encompasses an embryonic phase, which is restricted to the egg. In many taxa it is followed by a postembryonic phase, in which a larva hatches and passes through a series of larval phases, separated by molts. This developmental mode is referred to as ‘indirect development’. The larva differs significantly in morphology and life style from the adult and juvenile. Throughout this paper we will address individuals which have hatched from the egg shell but do not show the complete number of segments or differ strongly from juveniles in appendage morphology, as larvae [1]. The larval period in these species is followed by a juvenile (or ‘postlarva’) phase in which the adult morphology in respect of body segments, appendage number and morphology, is apparent but may differ from the sexually mature adult in size and proportion. The remaining malacostracan taxa possess an ontogenetic mode in which all pre-juvenile development takes place within the egg shell and an individual with adult-like morphology hatches from the egg. This is commonly referred to as ‘direct development’.

The nauplius is a planktonic larva with three pairs of functional appendages (mandibles, first and second antennae) that are together used for feeding and locomotion and is often considered being part of the ground pattern for Crustacea [2-5] or Tetraconata, assuming paraphyletic crustaceans [6]. Within malacostracans, however, only dendrobranchiate decapods and Euphausiacea possess a pelagic nauplius larva [7]. In these groups several nauplius stages are passed through, sequentially adding segments to the trunk. They are followed by stages with thoracic exopods functioning in propulsion, but without functional pleopods (dendrobranchiate *zoea*, euphausiid *calyptopis*) and stages with natatory pleopods (dendrobranchiate *postlarva*, euphauisiid *cyrtopia*) [1,8-14]. The majority of decapods possess an intermediate form of ontogeny in which more advanced pelagic, mostly planktotrophic, larval forms hatch and show correspondence to the dendrobranchiate *zoea*[15,16]. These larvae, which we will refer to as ‘zoea-like’ larvae, generally carry the complete- or nearly complete set of body segments, though the number and morphology of appendages differs strongly from the adult situation, as well as a paddle-shaped telsonic plate. There is considerable variation in number and morphology of larval stages in the Decapoda (see [4] for review). Stomatopods (with the exception of Lysiosquillidae) have so called *Pseudozoea*-larvae. In these pelagic larvae the head as well as the first and second thoracic segments bear appendages, while the pleomeres carry one pair of natatory appendages each [17,18].

Direct development is found in a large number of malacostracan lineages such as Leptostraca, Astacidea (Decapoda), Anaspidacea, Thermosbaenacea [19]. In these cases all of development takes place inside the egg shell (i.e. is embryonic) and ends with the hatching of the juvenile. Peracarida are generally regarded as direct developers [20-24]. This group has evolved a specialized mode of brood care, where eggs and larvae are carried in a ventral brood pouch (marsupium) until they are released. We classify the Mysidacea as larval- developers because hatching occurs early in development, and the inert larva, called ‘nauplioid’ with an incomplete set of segments but prominent first and second antennae [25,26], remains in the marsupium. This particular form of larval development is herein referred to as ‘pseudodirect development’, a term which has been recently introduced for development of Cladocera [27]. In certain other Peracarida, ie. in certain amphipods and isopods the free hatchlings also show aberration from adult morphology, e.g. the *pantochelis* and *protopleon* –stages of Hyperiida, and the *manca* of isopod [28,29].

**Figure 1:**
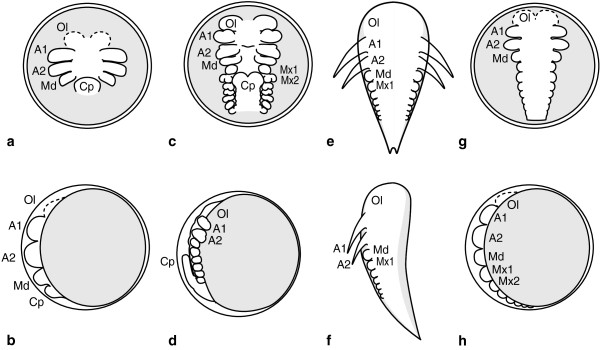
**Schematic overview of malacostracan germ band morphology in embryonic- and pseudodirect development. a**, **c**, **e**, and **g** represent ventral views of the germ band, **b**, **d**, **f** and **h** represent lateral views. **a**-**d** Simplified drawings of crayfish embryo modified from [68]. The general pattern applies to all Malacostraca with a yolk-free caudal papilla. **a** Ventral view of germ band at the egg-nauplius stage. Anlagen of the optic lobes, nauplius appendages and the caudal papilla are present. **b** Lateral view of same embryo. **c** Embryo at advanced, but still incomplete germ band stage. Optic lobe- and nauplius appendage rudiments are larger than posterior appendage anlagen. The caudal papilla is flexed anteriorly. **d** Lateral view of same embryo. **e** Early mysidacean nauplioid larva. An egg-membrane is missing. First and second antennae show advanced external morphology. Posterior appendage anlagen follow a gradual decrease in differentiation. **f** Lateral view of nauplioid larva. **g** Embryo representative of Peracarida except Mysidacea. The gap between naupliar- and postnaupliar appendage development is less distinct. The postnaupliar germ band displays gradual a/p -differentiation. The gradient is exaggerated in this drawing. **h** Lateral view of same embryo. Areas containing yolk are shaded grey in all drawings. Abbreviations: Ol optic lobes, Cp caudal papilla, A1, A2, Md, Mx1, Mx2, appendage anlagen. Yolk-rich areas are shown in light grey.

In a large number of malacostracan representatives with either direct development or an indirect developmental mode where the hatchlings are zoea-like larvae, the so called ‘egg-nauplius stage’ is traversed. It shows prominent appendage anlagen in the naupliar segments (first antennal-, second antennal and mandible buds). This is the result of a (more or less) synchronous formation of the naupliar appendage buds with a significant temporal advance compared to appendage anlagen of the following posterior segments (Figure 1a and -b). Whether the Mysidacea show temporal advance in development of the nauplius appendages is a matter of definition, since here the advance is seen only in the first and second antenna. We will consider it as advance in development of nauplius segments here. The so-called ‘nauplioid-larva’ of Mysidacea is sketched in Figure 1e and -f. The remaining Peracarida lack the clear temporal advance in nauplius appendage development. Instead they show only a slight difference in size between naupliar and postnaupliar appendage anlagen. Apart from that, segment differentiation along the anterior-posterior axis is continuous and follows a comparatively weak gradient (Figure 1g and -h) as demonstrated for amphipods and isopods [20,30,31]. In all representatives postnaupliar segment anlagen are formed subsequently as cells are proliferated in the teloblastic growth zone and can be distinguished by intersegmental furrows or appendage buds in the following embryonic stages. This growth zone is located in the preanal region of the germ band in all malacostracan representatives. In Leptostraca, Stomatopoda, Anaspidacea, Thermosbaenacea and Pleocyemata the preanal region is represented by a yolk-free posterior structure called the caudal papilla (Figure 1c and -d), which is flexed anteroventrally [32-41]. Here mesodermal units are proliferated from the mesoteloblast cells, and ectodermal units from the ectoteloblast cells, respectively.

Fritz Müller [42] was probably the first one who emphasized the evolutionary importance of the nauplius larva. Based on the discovery of the dendrobranchiate nauplius larva he suggested that all crustaceans evolved from a nauplius larva like ancestor, which implies that the nauplius as a larval stage represents a recapitulation of an adult stage. Jägersten [43] also stressed the importance of the nauplius larva. He suggested the nauplius larvae as the ‘primary larvae’ of all arthropods directly derived from a trochophora and that characters of the adults were transferred to the larva by a process he called “adultation” (which is quite the contrary from Müller’s ideas). Anderson [44] formulated a model of ancestral crustacean development. According to him development in the crustacean ground pattern comprised the formation of a nauplius larva bearing three functional pairs of appendages and four undifferentiated postnaupliar segment anlagen. Advanced larval forms or direct development are interpreted as derived in his view. The potentially primitive nature of a nauplius larva was further suggested by interpreting the situation in embryogenesis with advanced and synchronous development of the naupliar appendages, as described above, as a transient nauplius-phase in embryonic development – the so-called egg nauplius (Figure 1a and -b). The nauplius either as free-swimming larva or as egg-nauplius has been interpreted as representing a crustacean phylotypic stage [45-47], meaning that development is constrained to form a nauplius morphology in the early germ band as a prerequisite to subsequent morphogenetic processes in all members of the clade. The validity of such a concept must be questioned [48-50], if only because a nauplius/egg-nauplius stage is missing in some Malacostraca. Scholtz [7] argued that, based on correspondences between the nauplius larva and the egg-nauplius, the latter can be viewed as a developmental stage which is recapitulated: “*The egg-nauplius represents clearly a Müllerian (Haeckelian) recapitulation in the modern sense of an ancestral information (not necessarily one of an adult stage) that has been conserved and which is expressed during development*” [7], 182p. In this view the developmental program responsible for constructing a nauplius larva is still active in embryogenesis of species which have lost the larval stage. Furthermore he argues that the egg-nauplius stage is plesiomorphic for Malacostraca and that the free living nauplius larvae of dendrobranchiates and euphausiaceans have evolved independently to non-malacostracan nauplius larvae, as phylogenetic hypotheses of Malacostraca always place these groups in a nested position within the tree [5,19,51]. Scholtz [7] also suggests that the egg-nauplius facilitated secondary evolution of the free swimming nauplius larva of dendrobranchiates and euphausiaceans. He argues that from the starting point of an egg-nauplius stage only few evolutionary changes are necessary to generate a free swimming nauplius larva, compared to a starting point in which no egg-nauplius is present. The case of the egg-nauplius raises the question to what extent development must be modified during evolution to transform larval ontogeny into embryogenesis and vice versa. If the malacostracan egg-nauplius truly represents a transient larval stage in a recapitulatory sense (i.e. a larval stage which is now embryonized), then the developmental advances should comprise more than just external morphology. Hereafter we will use egg-nauplius as a descriptive term for embryonic semaphoronts externally characterized by the advanced three first appendage buds in the epidermis, but without implying recapitulation of a nauplius larva.

The problem inherent to the recapitulatory egg-nauplius concept is the lack of detailed morphological data that can be drawn from the observation of such early embryonic stages, as knowledge of tissue differentiation at a cellular level, especially within the mesoderm, is still scarce. Also, if heterochrony is a possible evolutionary mechanism involved in transformation of a nauplius larva or egg-nauplius, the complete developmental trajectories in naupliar- and postnaupliar tissues must be taken into account when comparison between species is performed. We have chosen to put muscle tissue to the focus of our ontogenetic study. Muscles form functional units together with the more readily observable epidermis/cuticle. Muscle patterns can therefore be assumed to evolve together with the structural capacity to perform certain body movements and behavioral features. That is why muscle development is particularly well suited to address questions of larval recapitulation and the applicability of the egg-nauplius concept. Here we present the first attempt to study evolution of malacostracan larval developmental features using muscle morphology.

Our previous work on muscle development of the crayfish *Procambarus fallax f. virginalis*[52] has revealed abundant data that can now be utilized to compare muscle development between taxa and reconstruct the evolutionary history of myogenesis. Here we apply a comparative ontogenetic approach to five malacostracan species, extending the methodology used on the crayfish, to obtain the temporal sequence of myogenic events. Our aim is (i) to describe developmental patterns of myogenesis for all five taxa, (ii) to test the consistency of muscle developmental patterns across taxa and (iii) by comparison draw first conclusions on the evolutionary history of myogenic sequences and their relation to the concept of a cryptic nauplius stage (a recapitulatory egg-nauplius). We believe that a detailed spatiotemporal study of muscle tissue patterning and differentiation during embryogenesis can reveal the extent to which the egg-nauplius is anchored to the developmental system and possibly uncover further heterochronic developmental traits that may be related to larval development.

**Figure 2:**
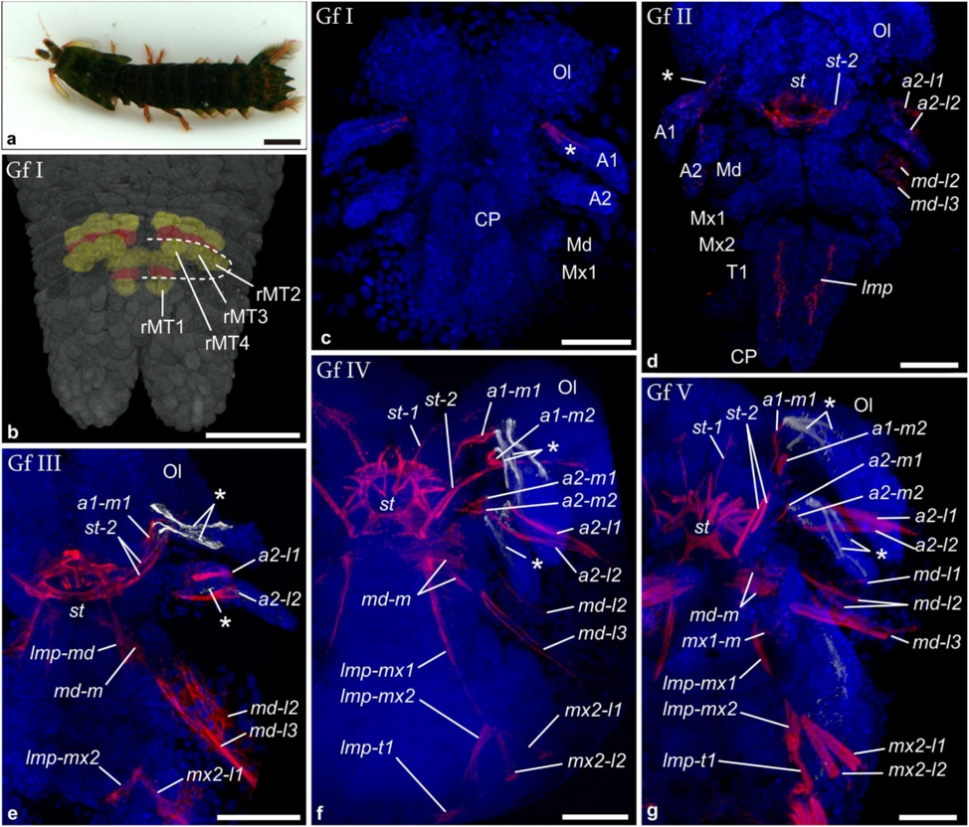
**Muscle ontogeny of *Gonodactylaceus falcatus. *a** Macroscopic image of adult female. **b**-**g** Maximum intensity projections of confocal image stacks taken from whole mount fluorescent staining. Nuclear stain (SYTOX) is shown in blue; muscle stain (Myo16-C6) is shown in red **(c-g)**. **b** blend projection of reconstructed nuclei with overlaying transparent image of ectodermal nuclei (grey). Dorsal view of **Gf I** caudal papilla. Mesoteloblasts (labeled for the right hemisegment: rMT1-rMT4) and two rows of mesodermal cells are reconstructed and highlighted in artificial colors alternating yellow and red. Mesoderm anlagen of more anterior segments show advanced proliferation which makes delineation of segments difficult. They are not highlighted. Mesoteloblasts form a ring in the caudal papilla. The dotted line marks the border between mesoteloblasts and mesodermal cells for the right hemisegment. **c**-**g** Ventral views of semaphoronts **Gf I** - **Gf V**. In **e**-**g** only the anterior left hemisegments are shown. **c Gf I** the external egg-nauplius morphology is apparent but the first maxilla bud is visible and the caudal papilla is elongate and flexed anteriorly. Intrinsic muscle precursor in antenna 1 is marked with an asterisk. **d Gf II**. *st*, *st-2*, *a2-l1*, *a2-l2*, *md-l2*, *md-l3* and *lmp* are visible (a clear distinction between *lmp-t1* and *lmp-post* cannot be made). Intrinsic muscle precursors of the first antenna appear (asterisk). **e Gf III**. *a1-m1*, *mx2-l1*, *lmp-md* and *lmp-mx2* appear. Intrinsic muscle precursors of the second antenna appear (asterisk). **f Gf IV**. *st-1*, *lmp-mx1*, *a1-m2*, *a2-m1*, *a2-m2* and *mx2-l2* are shown. *lmp-md* is no longer visible. **g Gf V**. Muscle precursors are slightly enlarged and *md-l1* has appeared. Abbreviations: Ol Optic lobes, Cp Caudal papilla, A1, A2, Md, Mx1, Mx2, T1 appendage anlagen. Scalebars are 1 cm in **a**, 50 μm in **b**, 100 μm in **c**-**g**.

**Figure 3:**
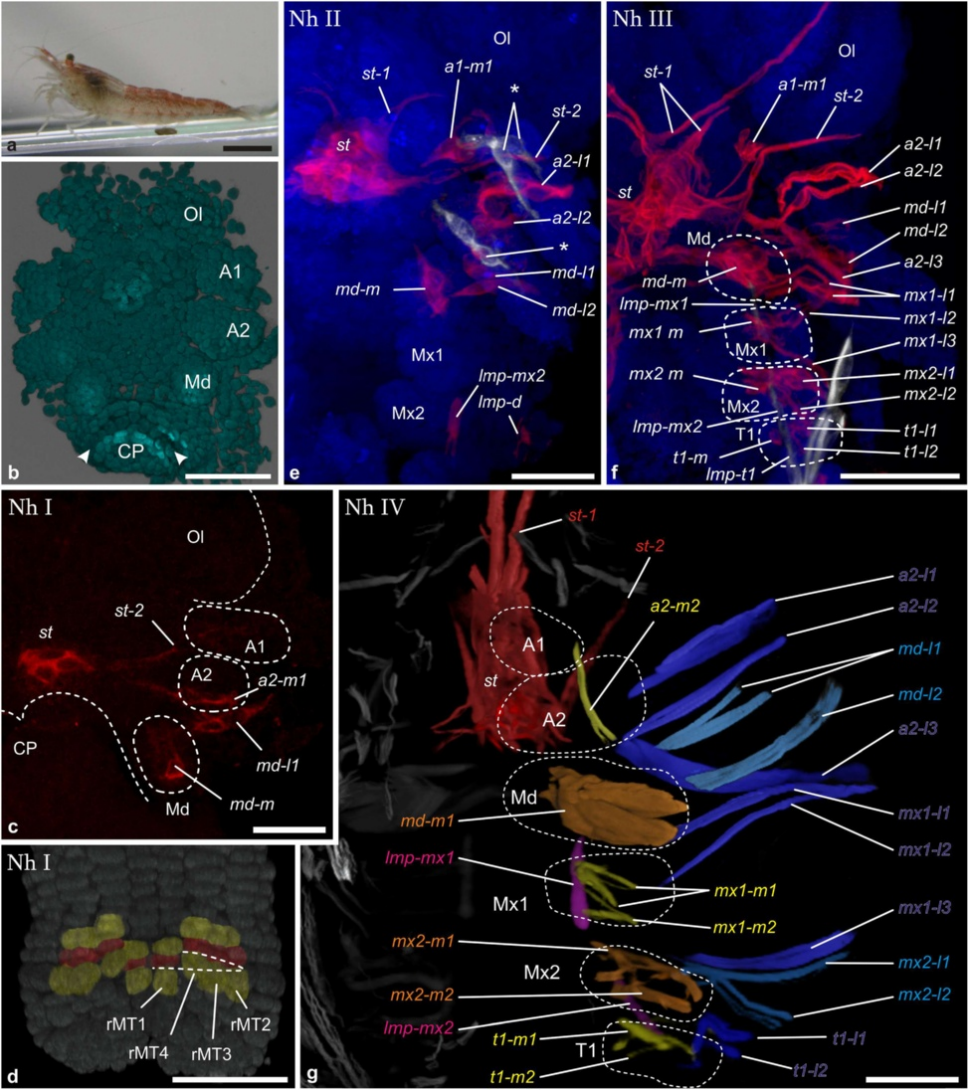
**Muscle ontogeny of *Neocaridina heteropoda. *a** Macroscopic image of adult. **b** and **d** blend-projection-, **c**-**g** maximum intensity projections of confocal image stacks taken from whole mount fluorescent staining. Nuclear stain (TOPRO-3): cyan **(b)**, grey **(d)**, blue **(e-f)**; muscle stain (Myo16C6-Cy3): red **(c, e and f)**; muscle stain (phalloidin-ALEXA564 ): multiple artificial colors **(g)**. **b** Egg-nauplius stage. Arrowheads mark the ectoteloblast row in the caudal papilla. **c**-**g** Ventral views of semaphoronts **Nh I** - **Nh IV**. In **c** and **e**-**g** only left body half is shown. **c Nh I**. Postnaupliar appendage anlagen are not seen but the caudal papilla is elongated and flexed anteriorly. Muscle precursors *st*, *st-2* and *a2-m1*, *md-l1* and *md-m* are present. **d** Dorsal view of **Nh I** caudal papilla (as in Figure 2b). **e Nh II**. *st-1. a1-m*, *a2-l1*, *a2-l2*, *mdl2* are present, as well as anterior longitudinal muscle precursors *lmp-mx2* and *lmp-d* . Intrinsic muscles appear (asterisks). **f Nh III**. Novel lateral extrinsic precursors: *a2-l3*, *mx1-l1*, *mx1-l2*, *mx1-l3*, *mx2-l1*, *mx2-l2*, *t1-l1* and *t1-l2*. Novel medial extrinsic precursors: *mx1-m*, *mx2-m* and *t1-m*. Novel longitudinal muscle precursor: l*mp-mx1*. **g Nh IV**. Individual precursors or groups of precursors are reconstructed and assigned artificial colors for better orientation. The color code is also applied to the labels. *a1-m* is no longer seen*. lmp-t1* is not shown. Medial precursors: *a2-m2*, *mx1-m1*, *mx1-m2*, *mx2-m1, mx2-m2, t1-m1* and *t1-m2*. Abbreviations: Ol optic lobes, Cp caudal papilla, A1, A2, Md, Mx1, Mx2, T1 appendage anlagen. Scalebars are 5 mm in **a**, 50 μm in **b**–**e,** 100 μm in **f** and **g**.

**Figure 4:**
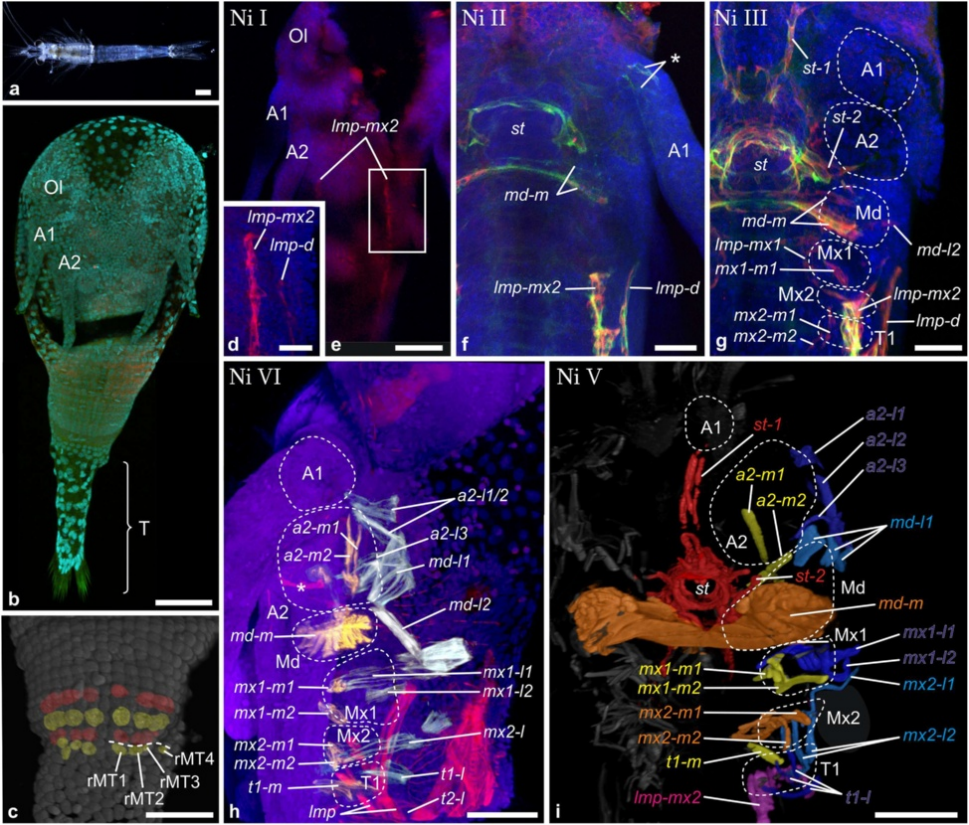
**Muscle ontogeny of *Neomysis integer. *a** Macroscopic image of adult. **b** Blend-projection, **c**-**g** maximum intensity projections of confocal image stacks taken from whole mount fluorescent staining. Nuclear stain (TOPRO-3): cyan **(b)**, blue **(d-h)**; muscle stain (anti- Myo16C6-Cy3): red **(d-h)**; Muscle stain (phalloidin-ALEXA488): green **(f, g)**, multiple artificial colors **(h, i)**. **b** Early nauplioid larva. The telson anlage is marked with brackets. **c** Ventral view of the growth zone (as in Figure 2b and 3d). Mesoteloblasts and mesodermal cells form transverse rows instead of rings. **d Ni I**. Anterior portion of *lmp* (*lmp-mx2*) and *lmp-d* is visible. **e** Overview of same specimen. White rectangle marks the field shown in **d**. **f** and **g** show anterior left half of the larva. **f Ni II**. *st*, *md-m* and intrinsic musculature (asterisk) have appeared. **g Ni III**. *st-1*, *st-2*, *md-l2*, *lmp-mx1*, *mx1-m1*, *mx2-m1* and *mx2-m2* are seen. **h Ni IV**. Lateral view; lateral and medial precursors are reconstructed and assigned artificial colors white and yellow respectively, the remaining muscle signal is shown in red. Novel lateral precursors: *a2-l1/2*, *a2-l3*, *md-l1*, *mx1-l1*, *mx1-l2*, *mx2-l* and *t1-l*. Novel medial precursors: *a2-m1*, *a2-m2, mx1-m2* and *t1-m*. **i Ni V**. Reconstructed precursors and precursor groups, as in Figure 3g. Novel lateral precursors: *a2-l1*, *a2-l2*, *a2-l3* (derived from *a2-l1*/*2*). *md-l2* is lost. Abbreviations: Ol optic lobes, A1, A2, Md, Mx1, Mx2, T1, appendage anlagen, T telson anlage. Scalebars are 2 mm in **a**, 100 μm in **b**, **e**, **h** and **I**, 50 μm in **c**, **f** and **g**, 25 μm in **d**.

**Figure 5:**
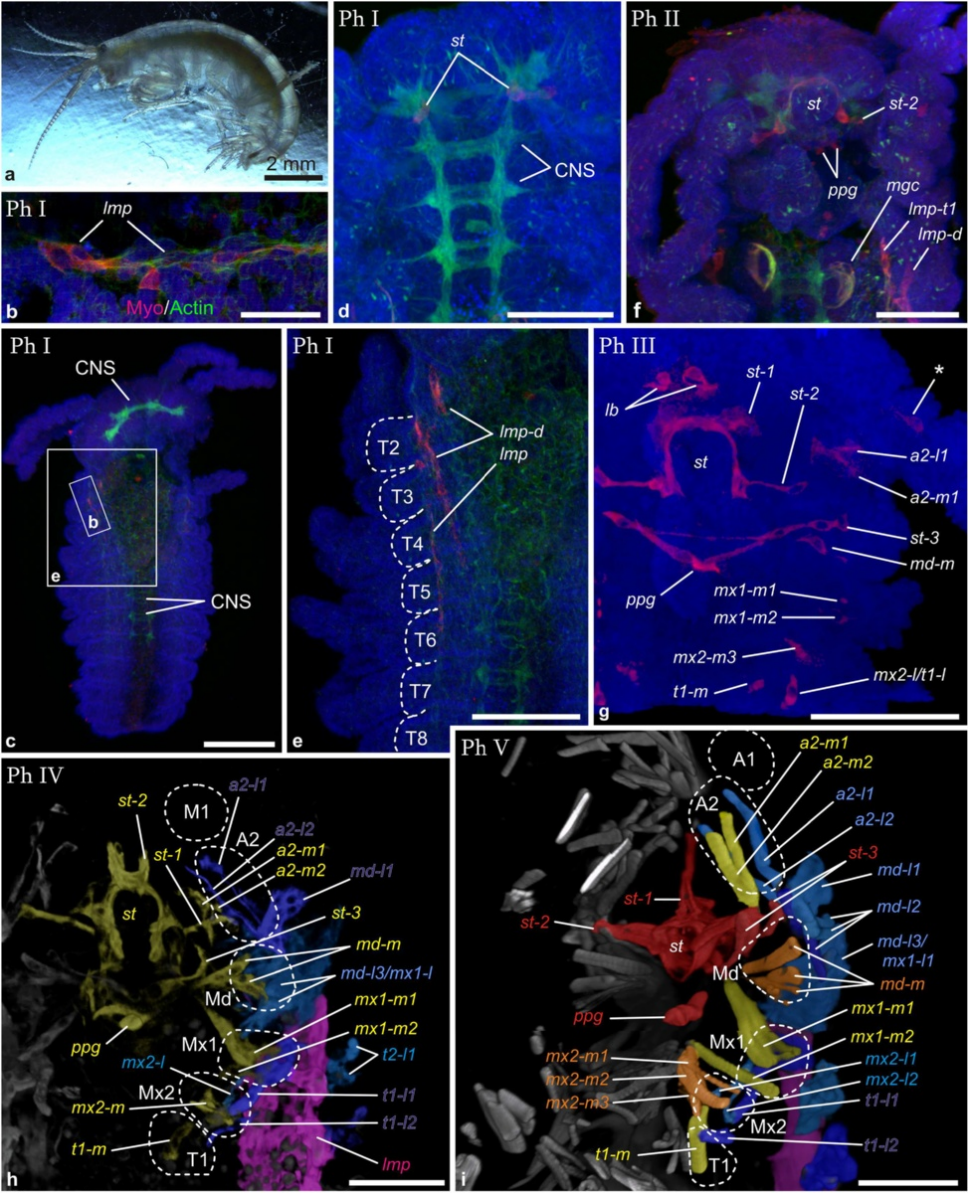
**Muscle ontogeny of *Parhyale hawaiensis. *a** Macroscopic image of adult. **c**-**g** Maximum intensity projections of confocal image stacks taken from whole mount specimens. **h** and **i** blend projections. Nuclear stain (TOPRO-3): blue in **b**-**g**. Muscle staining (anti- Myo16C6-Cy3): red in **b**-**g**, multiple artificial colors in **h**; muscle stain (phalloidin-ALEXA488): green in **b**-**f**, multiple artificial colors in **i**. **b** Extended optical section showing anterior portion of *lmp* (Overview shown in **c**). **c Ph I**. Dorsal view of embryo expressing phalloidin-signal in the CNS and first myogenic signals. Ventral portions of CNS are partly blocked by yolk. **d** Magnification of cephalic region in **Ph I**, ventral view. The CNS is shown as well as muscle pioneers of *st*. **e** Magnification of **c**, only left body half, dorsal view, showing *lmp* and *lmp-d*. Thoracic segments are marked with dotted lines. **f Ph II**. *st2*, *ppg*, *lmp-t1* and *lmp-d* are present. Midgut caeca muscle anlagen (*mgc*) are shown. **g**-**i:** ventral views of the anterior left embryonic region. **g Ph III**. Novel precursors: *lb* (labral muscle precursors), *st-1*, st-3, *a2-l1*, *mx2/t1-l*, *a2-m1*, *md-m*, *mx1-m1*, *mx1-m2*, *mx2-m3* and *t1-m*. **h** and **i** Individual precursors or groups of precursors are reconstructed and assigned artificial colors as in Figure 4i. **h Ph IV**. Novel precursors: *a2-l2*, *md-l1*, *md-l3/mx1-l*, *mx2-l*, *t1-l1* and *t1-l2,* and *a2-m2*. **i Ph V**. Additional precursors: *md-l2*, *mx2-l1*, *mx2-l2*, *mx2-m1*, *mx2-m2* and *mx2-m3*. Abbreviations: *A1*, *A2*, *Md*, *Mx1*, *Mx2,* T1 appendage anlagen, T2, T3, T4, T5, T6, T8 thoracic tergite anlagen. Scalebars are 2 mm in **a**, 25 μm in **b**, 100 μm in **c**-**i**.

Development of muscle patterns using genetic tools has only been studied on the amphipod *Parhyale hawaiensis*[53] and using histochemistry- and immunohistochemistry-techniques on dendrobranchiates [54,55], the American lobster *Homarus americanus*[56], the marbled crayfish *Procambarus fallax f. virginalis*[52], the amphipod crustacean *Orchestia cavimana*[57] and two isopod species *Porcellio scaber* and *Idotea baltica*[58]. The species investigated here comprise the stomatopod *Gonodactylaceus falcatus* (FORSKÅL, 1775) (Figure 2a) the decapod *Neocaridina heteropoda* (KEMP, 1918) (Figure 3a) and two peracarids: the mysidacean *Neomysis integer* (LEACH, 1814) (Figure 4a) and the amphipod *Parhyale hawaiensis* (DANA, 1853) (Figure 5a). Gross development of stomatopods is known from few studies using histology and external morphology [17,18,59,60]. *N. heteropoda* is a Southeast-Asian freshwater shrimp and a popular pet for aquarists. Postembryonic ontogeny has been described for related species [61,62]. *Neomysis integer* has been subject to previous studies of germ band development [26] and other aspects of ontogeny [25,63]. *Parhyale hawaiensis* has recently become a popular model organism for developmental biology and is cultured in several laboratories around the world [64].

## Methods

Egg material of *Gonodactylaceus falcatus* was collected from females caught in the wild on Coconut Island (Hawai‘i Institute of Marine Biology) in September 2008. Animals were kept in a large tank under a continuous flow of fresh sea water. Eggs were collected from gravid females in periods of approximately 12 h and preserved for further investigation. Egg material of *Neocaridina heteropoda* was collected from the pleopods of gravid females of an established lab culture in the facilities of the zoology department at Rostock University. Collection was performed at time periods of approximately 24 h. Embryos were freshly dissected from the egg shells in PBS (1.86 mM NaH_2_PO_4_, 8.41 mM Na_2_ HPO_4_, 175 mM NaCl, pH 7.4). Gravid females of *Neomysis integer* were caught from the south pier of the Rostock harbor between April and August of 2011. Eggs or nauplioid larvae were removed from the marsupium and kept in artificial sea water (15PSU) for further processing. Animals could not be reared successfully but material covering all developmental stages could be collected from a larger batch of females. Egg material of *Parhyale hawaiensis* was collected from the marsupium of female animals kept in a permanent lab culture at Rostock University (original stock from Anastasios Pavlopoulos, MPI CBG Dresden). Embryos were freshly dissected as described above for *Neocaridina heteropoda*.

### Staining and microscopy

Fixation of dissected embryonic material was carried out by incubation in 4% paraformaldehyde (Electron Microscopy Sciences)/PBT (PBS with 0.3% triton X-100 as detergent). Incubation times varied between 30′ and 60′ and were adapted for the individual developmental stage. Larvae of *N. integer* were opened dorsally with tungsten needles to acquire full exposure of tissues to the fixative and staining solutions. Immunohistochemical staining was applied following standard protocols as described previously [52]. Monoclonal antibody 16C6 which is reactive to myosin-heavy chain [56,58] was used to label early muscle progenitors at 10× dilution. 10% ROTI-Block (Carl Roth GmbH, Karlsruhe) was used as blocking agent in the rinsing- and staining solutions. For late developmental stages 1.5% DMSO (AppliChem, Darmstadt) was added to all solutions to increase tissue permeability, along with 0.3% BSA (Merck, Darmstadt). Incubation times were adapted individually for large specimens. Goat-AffiniPure anti-mouse IgG H + L labeled with Cy3 (Jackson Immunoresearch) was applied for antibody detection.

F-Actin was labeled histochemically with ALEXA-488- or ALEXA-561 conjugated phalloidin (Invitrogen Molecular Probes) following the manufacturers protocols. Nuclear staining was performed using TOPRO-3 (Invitrogen Molecular Probes) or SYTOX (Invitrogen Molecular Probes). CELL MASK (Invitrogen Molecular Probes) was used as unspecific tissue stain on *Gonodactylaceus falcatus*. All samples were mounted in Vectashield (Vector Laboratories, Burlingame CA) for microscopy.

Confocal image stacks were recorded with a Leica DMI6000 CFS confocal laser scanning microscope, equipped with a conventional scanning system Leica TCS SP5 II. Step sizes between successive scanning planes ranged from 0.4 to 1.0 μm. Volume Data were calculated from confocal stacks and edited using IMARIS 7.0 (Bitplane, Switzerland). Editing included manual reconstruction of volume partitions (e.g. mesoteloblast cells or muscles at advanced developmental stages) and assignment of individual colors to improve clarity for complex structures. Image tables were created in COREL DRAW X3. Micrographs of adult specimens of *N. integer* and *P. hawaiensis* were recorded using a DISCOVERY V12 stereo microscope equipped with an AxioCam ICc 3 – Camera (Carl Zeiss, Jena). Images were edited in COREL PHOTOPAINT X3.

Laboratory work on any of the crustacean species which are part of our study does not raise ethical issues. Therefore approval from a research ethics committee is not required.

### Myogenesis terminology

We apply a terminology adapted from the *founder cell model* of myogenesis proposed for insect development [65,66]. Spindle shaped mononucleate cells which stain positive for muscle specific proteins (in our case myosin-heavy chain) are termed *muscle pioneer cells*. According to the model these cells serve as scaffold for muscle formation. Fusion of surrounding mesodermal cells (myoblasts) leads to multinucleate units, also detectable with myosin and phalloidin. These units are called *muscle primordia*. The term *muscle precursor* refers to muscle pioneer cells and muscle primordia likewise. The scaffolding role of the muscle precursor pattern implies that parts of the initial pattern can be lost during development as observed in grasshoppers [65]. Therefore it is not possible to assign a certain muscle pioneer or precursor to a specific adult muscle or function in every case. We use a modified version of the terminology presented in [56] to specify individual precursors. The terms relate only to the position and orientation of muscle precursors within the embryo and do not immediately imply adult function or even homology between species. A list of all precursors is given in Table 1.

**Table 1:** The table gives a list of all muscle precursors described and discussed throughout the paper

**Body region**	**Muscle precursor group**	**Appendage anlage/segment**	**Muscle precursor**
Stomodeal region	Stomodeal precursor group	-	*st*
		-	*st-1*
		-	*st-2*
		-	*st-3*
		-	*ppg*
Appendage anlagen (distal region)	Intrinsic precursors	-	*Intr*
Appendage anlagen (proximal region)	Medial extrinsic precursors	A1	*a1-m*
			*a1-m1*
			*a1-m2*
		A2	*a2-m*
			*a2-m1*
			*a2-m2*
		Md	*md-m*
		Mx1	*mx1-m*
			*mx1-m1*
			*mx1-m2*
		Mx2	*mx2-m*
			*mx2-m1*
			*mx2-m2*
			*mx2-m3*
		T1	*t1-m*
			*t1-m1*
			*t1-m2*
	Lateral extrinsic precursors	A2	*a2-l1/2*
			*a2-l1*
			*a2-l2*
		Md	*md-l1*
			*md-l2*
			*md-l3*
			*md-l3/mx1-l1*
		Mx1	*mx1-l1*
			*mx1-l2*
			*mx1-l3*
		Mx2	*mx2-l/t1-l*
			*mx2-l1*
			*mx2-l2*
			*mx2-l3*
		T1	*t1-l1*
			*t1-l2*
Trunk region	Longitudinal precursors (*lmp*)	Md	*lmp-md*
		Mx1	*lmp-mx1*
		Mx2	*lmp-mx2*
		T1	*lmp-t1*
		All segments (dorsal region)	*lmp-d*
		Growth zone/telson	*lmp-post*

Muscle precursor terms represent a code of letters and numbers, given in italics, which relate to the body region (e.g. stomodeum *st*) appendage anlage or body segment (e.g. first antenna *a1*). It also refers to the anteroposterior arrangement of precursors from anterior to posterior (numbers). Muscle precursors are sorted into groups by appendage/segment affiliation (A1, A2, etc.), muscle precursor group (stomodeal precursor group, intrinsic appendage muscle precursors, medial extrinsic appendage muscle precursors, lateral extrinsic appendage muscle precursors, longitudinal trunk muscle precursors), and body region (stomodeal region, distal region of appendage anlage, proximal region of appendage anlage, trunk region). Certain precursors (*a1m*, *mx1-m*, *mx2-m* and *t1-m*) give rise to multiple precursors found at later stages (e.g. *a1-m* gives rise to *a1-m1* and *a1-m2*) but the way this is achieved is uncertain (there are three possibilities: a precursors splits into two precursors, an additional precursor arises at a more anterior position, or an additional precursor arises at a more posterior position). Three precursors (*a2-l1/2, md-l3/mx1-l1, mx2-l/t1-l*) give rise to two different muscle units each (*a2-l1*, *a2-l2*; *mx2-l*, *t1-l* and *md-l3*, *mx1-l1*, respectively).

We define the following muscle precursor groups following [67]:Muscle precursors are given in italics throughout this paper.

‘*stomodeal muscle precursors*’

Precursors of ring- and dilatator muscles associated with the stomodeum.

‘*intrinsic appendage muscle precursors*’

Muscle precursors located within the appendage anlage and extending distally from the coxa. They function in moving podomeres of the appendage in respect to each other.

‘extrinsic appendage muscle precursors’

Extrinsic muscles serve to move the coxa of the appendage in respect to the trunk.

‘medial extrinsic appendage muscle precursors’

Muscle precursors extending medially into the trunk from the proximal region of the appendage anlage.

‘lateral extrinsic appendage muscle precursors’

Muscle precursors extending laterally into the trunk from the proximal region of the appendage anlage.

‘longitudinal muscle precursors’

Muscle precursors which extend in anterior-posterior direction within the trunk.

### Semaphoront specification and ontogenetic sequences

We avoid established staging nomenclature (as given for example for *P. fallax f. virginalis* in [68]). Instead we will apply a numerical code using roman numbers to specify an individual at the respective time of its life, hereafter referred to as ‘semaphoront’ [69]. The code does not imply any correspondence between taxa and only refers to the semaphoronts in relation to each other. Emergence of novel muscle pioneer cells or muscle precursors represent the criteria by which these semaphoronts are specified. The development of embryonic musculature in the crayfish *Procambarus fallax f. virginalis* was described earlier [56]. Developmental stages St3, St5, St7 and St9 described therein are utilized for comparison here and specified as semaphoronts **Pf I, Pf II, Pf III** and **Pf IV** respectively.

## Results

In the following section semaphoronts are listed and described (roman numbers). All description refers to the hemisegments of the left body half in ventral view. Our investigation focuses on two body regions which we find to be most enlightening in respect of myogenic evolution: the anterior six segments of the trunk and the telson anlage. Therein we concentrate on the muscle precursor groups listed above. This excludes visceral- and heart-musculature. Intrinsic muscles of the appendages are also largely excluded from our discussion for reasons of clarity, but are considered in early developmental stages. In the caudal papilla (if present), the posterior pleon segments and the telson anlage we focus on longitudinal muscle precursors (Table 1). The position of mesoteloblast cells and early mesodermal segmental units are recorded as well.

**Figure 6:**
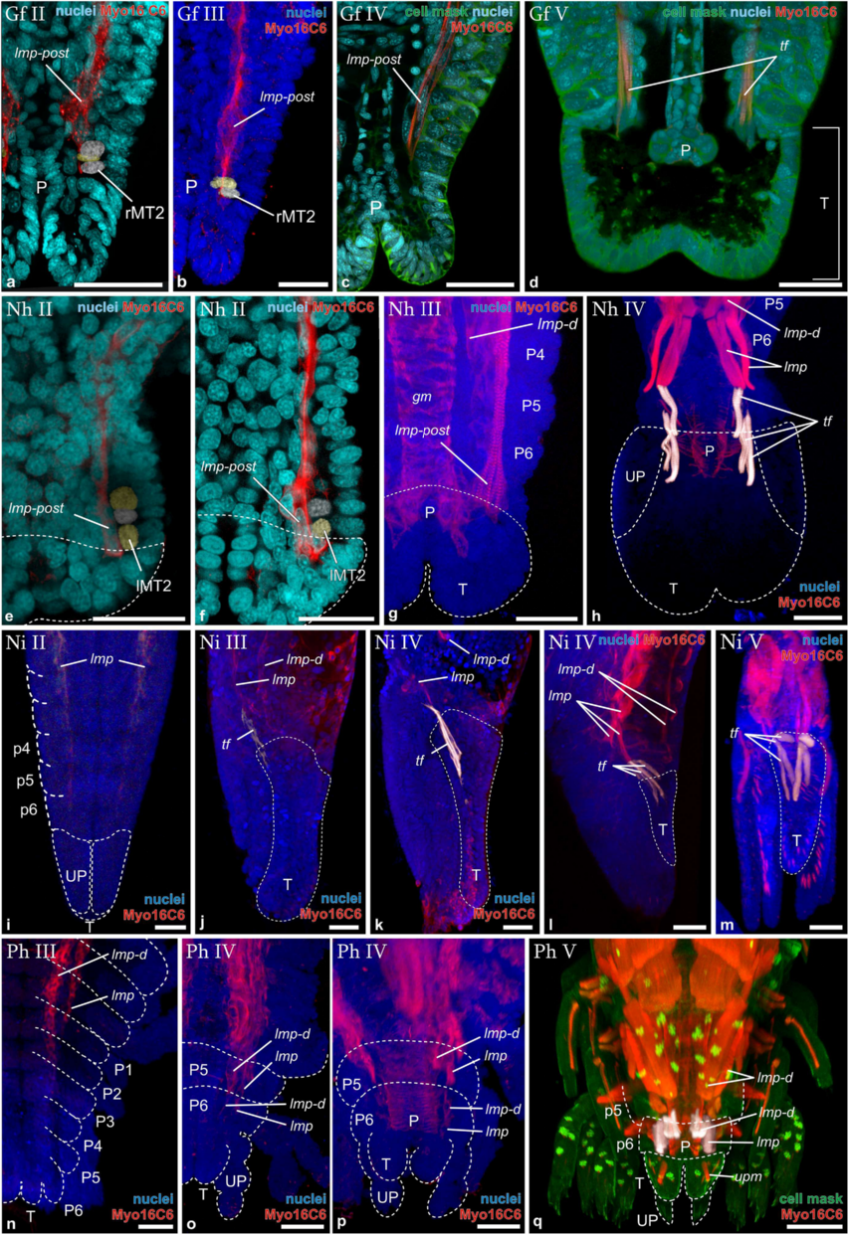
**Muscle ontogeny in the caudal papilla, posterior pleon- and telson anlage of all four species. a**-**f** extended optical sections, **g**-**q** maximum intensity projections. **a**-**h**, **n**-**q** dorsal view, **i** ventral view, **j**-**l** lateral view, **m** dorsolateral view. Anterior is oriented to the top in all images, **a**-**c**, **e**-**g**, **n** and **o** show only the right body half. **a Gf II***. lmp-post,* mesoteloblast rMT2 and reconstructed mesodermal cells are shown. **b Gf III***. lmp-post is enlarged*, mesoteloblasts and mesodermal cells are visible. **c Gf IV***.* Broad shape of *lmp-post*. Mesoteloblasts and mesodermal cells are no longer visible. **d Gf V***.* Telson is delineated (bracket). *tf* inserts in the anterior dorsal region of telson. **e Nh II***. lmp-post*, mesoteloblasts and mesodermal cells are visible. Telson is delineated. **f Nh II** slightly later in development (specimen not shown in Figure 3)*. lmp-post*, has enlarged, only one mesodermal cell row can be identified. Telson is delineated. **g Nh III***.* Broad shape of *lmp-post,* striation is visible. Mesoteloblasts and mesodermal cells are no longer visible. *lmp-d* is visible in P4. Telson is delineated. **h Nh IV***. tf* show the adult tripartite pattern and insert anterodorsally in the telson. **i Ni II***.* Telson and uropods are delineated. Mesoteloblasts and mesodermal cells are no longer visible. *lmp* shows a-p gradient of differentiation and reaches P6. **j Ni III** Posterior part of *lmp* (*tf*) extends into telson. **k Ni IV***. tf* is enlarged and separated from *lmp*. **k Ni IV**. Slightly later in development (specimen not shown in Figure 4)*.***l Ni IV**. Yet later in development: *tf* show tripartite structure. **m Ni V**. *tf* show adult morphology and insert anterodorsally in the Telson. **n Ph III***.* Telson and uropods are delineated. Mesoteloblasts and mesodermal cells are no longer visible. *lmp* shows a-p gradient of differentiation and reaches P1. *lmp-d* reaches P3. **o Ph IV***. lmp* and *lmp*-*d* are enlarged and show metameric subdivision. Both reach P6. **p Ph IV**. Slightly later in development (specimen not shown in Figure 5). Individual segmental muscle units are enlarged and gut muscle becomes apparent. **q Ph V**. *lmp* and *lmp-d* are differentiated in P6, but telson remains free of musculature. The muscle precursors marked *upm* lie within the uropods, ventrally of the telson. Abbreviations: P proctodeum, rMT2 second mesoteloblast cell of left body half, *tf* telson flexor muscles, T telson anlage, UP uropod anlagen, *upm* uropod muscle precursors, P1-P6 pleon segments 1–6, *gm* gut muscle primordia. Scalebars are 50 μm in all panels.

*Gonodactylaceus falcatus* (Figure 2a):

**(Gf I)** The first muscle signals could be detected in intrinsic muscle precursors of the second antenna anlage after the egg-nauplius stage. At this developmental stage unsegmented rudiments of the first and second antenna, the mandible and the first maxilla are visible while the second maxilla bud at this stage is not yet visible (Figure 2c). In the caudal papilla the undifferentiated anlagen of the thoracic segments are present. Figure 2b shows the mesoteloblasts and two rows of mesoteloblast progeny. **(Gf II)** The muscle progenitor complex of semaphoront **Gf II** is characterized by two lateral extrinsic muscle primordia (*a2-l1*, *a2-l2*) associated with the second antenna and two lateral extrinsic precursors (*md-l2*, *md-l3*) associated with the mandible anlage (Figure 2d). Around the developing stomodeum a ring-like assembly of muscle forming cells (*st*) has formed. From the posterolateral margin of *st* another precursor (*st-2*) extends anterolaterally and dorsally. Longitudinal muscle primordia (*lmp*) form a ventral strand which extends from the first thoracic segment into the caudal papilla. The posterior end of the longitudinal precursor strand (*lmp-post*) is located just medially of the lateral-most mesoteloblast *MT2* (Figure 6a), which can be specified by the characteristic cell shapes and -arrangements in the growth zone mesoderm. Slightly anterior to the mesoteloblast the longitudinal strand contains a cluster of nuclei which show no trace of segmental order. **(Gf III)** As development proceeds, an extrinsic muscle primordium of the first antenna (*a1-m1*) arises and extends medially from the base of the appendage rudiment and posteroventrally towards the stomodeal muscle ring (*st*) (Figure 2e). A muscle primordium (*md-m*) has arisen in the mandible segment and a longitudinal muscle primordium (*lmp-md*) is attached to the posterolateral margin of *st*. The anterior end of the ventral longitudinal muscle strand can now be found in the second maxilla segment where it touches a novel lateral muscle primordium (*mx2-l1*). The posterior end of the longitudinal muscle strand has enlarged slightly and shows an increased number of nuclei. The cluster of nuclei within the strand appears more condensed anterior to the teloblasts (Figure 6b). **(Gf IV)** Yet later in development a novel muscle primordium (*st-1*) extends from the anterior margin of *st* anterodorsally (Figure 2f). An additional medial extrinsic muscle primordium (a*1-m2*) of the first antenna is now present and additional medial extrinsic primordia (*a2-m1, a2-m2*) have appeared in the second antenna segment. In the mandible segment the medial muscle primordium (*md-m*) is now composed of several units with transverse orientation. Interestingly the longitudinal muscle primordium of the mandible segment is no longer observable. However, a prominent longitudinal primordium (*lmp-mx1*) now extends throughout the first maxilla segment from the posterior of the medial mandible muscles. An additional lateral muscle primordium of the second maxilla segment (*mx2-l2*) can be seen. The posterior longitudinal muscle strand has increased in width and now shows striation. The mesoteloblasts are no longer visible as all body segments have been formed and cell division has proceeded within all mesodermal units at this time of development (Figure 6c). Segmental furrows have formed throughout the entire trunk (Additional file 1: Figure S1a). **(Gf V)** In the final stage of muscle development presented here an additional lateral extrinsic precursor has formed in the mandible segment (*md-l1*) and a medial muscle precursor of the second maxilla anlage is becoming visible (*mx1-m*) (Figure 2g). In the caudal papilla differentiation of the telson flexor muscles has begun, which insert dorsally and ventrally (not shown) at the anterior margin of the telson (Figure 6d).

*Neocaridina heteropoda* (Figure 3a):

No muscle primordia can be detected at the egg-nauplius stage (Figure 3b). **(Nh I)** The earliest detectable muscular pattern is found after the egg-nauplius stage, when the caudal papilla has elongated (Figure 3c). Appendage rudiments of the head segments up to the mandible are distinguishable, while the segments of the first and second maxillae are not yet fully differentiated. The caudal papilla, containing the mesoteloblasts and at this stage the early mesodermal units of the thoracic segments is folded ventrally and oriented anteriorly. The initial set of muscle precursors comprises the stomodeal muscle ring (*st*), a laterally extending precursor associated with the stomodeum (*st-2*) and a medial extrinsic muscle precursor of the second antenna (*a2-m1*). The latter is oriented from the second antennal base towards the stomodeum at this initial stage of morphogenesis. The mandibular segment contains one muscle precursor (*md-m*) extending medially and one (*md-l1*) extending laterally from the limb bud. In the caudal papilla the single mesoteloblasts can be identified (Figure 3d). Together with undivided mesoteloblast progeny they form a stereotypic pattern of segmentally arranged cell rings. **(Nh II)** One step later in muscle development of *N. heteropoda* intrinsic muscle primordia of the first and second antenna are visible (Figure 3e). Also an extrinsic muscle primordium (*a1-m1*) running medially from the first antenna bud towards the stomodeum is found. An anterior extension of the stomodeal muscle group is present (*st-1*), and the second antenna and mandible segment now each possess two lateral extrinsic primordia (*a2-l1*, *a2-l2*, *md-l1*, *md-l2*). *a2-m1* has moved to a position within the elongate second antenna-anlage, where it will give rise to intrinsic musculature (marked with an asterisk in Figure 3e). Finally longitudinal muscle precursors become visible in the segment of the second maxilla. The formed strand (*lmp-mx2*) extends into the caudal papilla. At the posterior end (*lmp-post*) a small accumulation of nuclei is visible medially to the mesoteloblasts (Figure 6e and -f). An additional parallel strand of longitudinal musculature (*lmp-d*) is being formed at a more dorsal position but it does not extend as far posteriorly as the ventral strand (Figure 3e). **(Nh III)** In semaphoront III the medial mandible muscle precursor *md-m* has now formed an extension that crosses the median region of the germ band (Figure 3f). Medial extrinsic precursors are now present associated with both maxillae- and the first thoracopod anlagen (*mx1-m, mx2-m, t1-m*) and also lateral extrinsic precursors have emerged in the same segments (*mx1-l1*, *mx1-l2*, *mx1-l3*, *mx2-l1*, *mx2-l2*, *t1-l1*, *t1-l2*). A longitudinal muscle primordium (*lmp-mx1*) is visible for the first time in the first maxilla segment. A third lateral extrinsic precursor of the second antenna has also become visible *(a2-l3*), which extends posterolaterally and crosses *md-l1* and *md-l2*. The posterior end of the dorsal longitudinal strand (*lmp-d*) has extended posteriorly into pleomere 4 (Figure 6g). The posterior end of the ventral longitudinal muscle strands (*lmp-post*) has enlarged and also the more anterior portions have obtained additional nuclei. The mesoteloblasts can no longer be seen, indicating that segment formation has stopped and the mesoteloblast progeny have proliferated into mesodermal cells of the mesodermal units (Figure 6g). The intersegmental furrows of the posterior pleon segments and the telson can be distinguished (Additional file 1: Figure S1b). **(Nh IV)** Finally, as adult morphology becomes apparent we find a new muscle primordium of the second antenna (*a2-m2*), which extends anteromedially from the posterolateral margin of the appendage base (*a2-m1* is no longer present outside of the appendage anlage). The first and second maxilla segments, as well as the first thoracopod segment now each contain two medial extrinsic precursors (*mx1-m1*, *mx1-m2*, *mx2-m1*, *mx2-m2*, *t1-m1*, *t1-m2*). In the developing telson the telson flexor muscles (*tf*) are present and extend anteriorly into the sixth pleomere (Figure 6h).

*Neomysis integer* (Figure 4a):

Mysid ontogeny differs from the species described above as a large part of development is confined to the nauplioid cuticle [63]. No muscle precursors can be detected in the embryonic stages. A small inert larva called ‘nauplioid’ hatches but remains in the marsupium (Figure 4b). The prominent uniramous first and second antennae are sheeted by cuticle and bear setae but posteriorly to them no appendage buds are visible. Yet the formation of segment anlagen in the nauplioid larva is comparatively advanced as can be seen from the regular arrangement of ectodermal and mesodermal cell material in the germ band. The mesoteloblast cells and early trunk mesoderm anlagen are arranged in transverse rows (not in rings, as in *G. falcatus* and *N. heteropoda*) (Figure 4c). **(Ni I)** The first myogenic signals in *N. integer* larvae are detected after all segments have been laid down and appendage rudiments are present in the entire trunk (not shown). The ventral longitudinal muscle strand extending posteriorly from the second maxilla segment (*lmp-mx2*), is the first muscular primordium to become visible (Figure 4d and -e), together with the dorsal longitudinal muscle strand (*lmp-d*) (Figure 4d, only anterior segments shown). The ventral longitudinal muscle strand extends from the second maxilla segment (*lmp-mx2*) to the anterior pleon segments, though the exact position of the posterior end is difficult to specify due to insufficient staining intensity at this early stage. The initial strands are continuous and are not separated into distinct segmental units. Yet internal segmentation can be seen later on and we will treat the longitudinal muscle precursors of the second maxilla- and first thoracopod-segments as discrete units for comparison in our discussion (see below). Also the posterior end of the germ band is free of muscular tissue at this stage (not shown) and remains free also in the second semaphoront. **(Ni II)** In semaphoront II the stomodeal muscle ring (*st*), the intrinsic muscles of the first antennae and the median muscle primordium of the mandible (*md-m*) have been added to the nauplioid muscle pattern (Figure 4f). Both longitudinal strands have increased in width. A gradual decrease in differentiation from anterior to posterior is apparent and the ventral strand reaches the sixth pleomere (only the ventral strand *lmp* is shown in Figure 6i). A posterior muscle precursor (*lmp-post*) associated with the growth zone, such as in *G. falcatus* and *N. heteropoda* is not found. Segmental furrows have formed throughout the entire trunk (Additional file 1: Figure S1c). **(Ni III)** The next observed myogenic events are the origination of the anterior and lateral stomodeal muscle precursors (*st-1*, *st-2*), together with a median muscle precursor in the first and second maxilla segment (*mx1-m1, mx2-m1, mx2-m2*) (Figure 4g). A lateral extrinsic muscle precursor appears in the mandible segment (*md-l2*). Also an additional longitudinal muscle precursor (*lmp-mx1*) has formed in the segment of the first maxilla. The longitudinal muscle strands have now reached the last pleon segment and extend even into the telson rudiment (Figure 6j). **(Ni IV)** The following semaphoront possesses two lateral extrinsic muscle primordia of the second antenna (*a2-l1/2, a2-l3*), the mandible (*md-l1, md-l2*), the first maxilla (*mx1-l1*, *mx1-l2*), as well as a single lateral extrinsic muscle primordium of the second maxilla (*mx2-l*) and the first thoracopod (*t1-l*) (Figure 4h). The second antenna has obtained medial extrinsic muscle primordia (*a2-m1, a2-m2*). The first maxilla- anlage now shows two medial muscle precursors (*mx1-m1*, *mx1-m2*) and a medial muscle primordium is now present in the first thoracic segment (*t1-m*). The posterior portion of the ventral longitudinal muscle strand (*lmp*) has differentiated into an elongate muscle precursor giving rise to the telson flexor muscles in the anteroventral region of the premature telson (Figure 6k and -l). **(Ni V)** After the larva has molted the nauplioid cuticle the first antenna still lacks extrinsic musculature (Figure 4i). The first lateral extrinsic muscle primordium of the second antenna *a2-l1/2* has become clearly separated into two distinct units (*a2-l1*, *a2-l2* ). Also an additional lateral extrinsic muscle precursor has emerged (*mx2-l2*) and one of the lateral extrinsic precursors associated with the mandible (*md-l2*) is no longer visible. The telson and uropods are clearly differentiated and the telson flexor muscles display the adult arrangement (Figure 6m).

*Parhyale hawaiensis* (Figure 5a)

Unlike in *G. falcatus* and *N. heteropoda*, muscle development in *P. hawaiensis* initiates at a relatively late developmental stage and is completed rapidly. Semaphoronts **(Ph I)**, **(Ph II)** and **(Ph III)** correspond to stage S22 following Browne et al. (2005), **(Ph IV)** corresponds to S24 and **(Ph V)** to S28 respectively. **(Ph I)** At the onset of myogenesis the germ band contains the complete set of segments, each with elongated appendage anlagen. The antennae and thoracic limbs are fully subdivided into final podomeres. Figure 5c shows an overview of an embryo at the earliest stage where muscle formation is detectable. F-actin which is marked by the green phalloidin signal in these early muscle primordia is restricted to the cell cortex and is not co-localized with myosin-signal in the initial muscle precursors (Figure 5b). F-actin staining also revealed that the central nervous system is developed to a large extent; showing paired ganglion anlagen (Figure 5d). The initial set of head muscles includes a pioneer cell located close to – and dorsally of the developing tritocerebral hemiganglion, on each side of the stomodeum. This muscle pioneer cell represents the anlage of the stomodeal muscle ring (*st*) and exhibits cytoplasmic protrusions which extend anteriorly. At the same time pioneer cells forming the dorsal and ventral longitudinal muscle strands (*lmp*, *lmp-d*) can be found in the first thoracic segment extending posteriorly (Figure 5e). The ventral longitudinal strands terminate in the 7th-, the dorsal longitudinal strands in the 6th thoracic segment. No metameric pattern can be recognized within the strands at this stage. **(Ph II)** The stomodeal muscle anlage (*st*) now encloses the stomodeum anteriorly (Figure 5f). A novel muscle pioneer *st-2* is connected to *st* at the posterolateral margin and extends laterally. Slightly posterior to the stomodeum a pair of pioneer cells forms yet another novel muscular unit which protrudes ventrally and inserts medially of the paragnaths. This muscular unit, which is not observed in any of the other investigated malacostracan species in this study, is termed ‘pharyngo-paragnathal muscle’ (*ppg*). The ventral longitudinal muscle strand primordium *lmp* is detectable from the first thoracic- to the second pleon segment (not shown). **(Ph III)** The following stage is characterized by the appearance of further muscle pioneer cells in the head region (Figure 5g). Intrinsic muscle pioneers are now present in both proximal podomeres of the second antenna. Also the anlage of the labrum now contains paired muscle primordia. A novel precursor (*st-3*) associated with the stomodeal muscles is observed in a transverse position posterolaterally of the stomodeum, which displays a thin cytoplasmic extension across the medial region of the germ band. Two medial and lateral extrinsic muscle primordia (*a2-m1*, *a2-l1*) associated with the second antenna can be seen. The medial mandible muscle anlage (*md-m*) is present, as well as additional medial extrinsic muscle primordia (*mx1-m1*, *mx1-m2, mx2-m3, t1-m*) associated with the respective appendage anlagen. One lateral extrinsic precursor (*mx2*/*t1-l*) is found which gives rise to extrinsic musculature of second-maxilla and first thoracopod. The posterior ends of *lmp* and *lmp-d* are detectable only anterior to the third pleon segment and first pleon segment respectively (Figure 6n). Segmental furrows have formed throughout the entire trunk (Additional file 1: Figure S1d). **(Ph IV)** Formation of additional lateral extrinsic muscle primordia in the remaining appendage anlagen from the mandible to the first thoracopod has occurred (*a2-l2, md-l1, md-l3/mx1-l*, *mx2-l*, *t1-l1, t1-l2*) (Figure 5h), but extrinsic primordia can be observed also in the following segments down to the sixth pleopod (not shown). An additional medial extrinsic muscle precursors associated with the second antenna has formed (*a2-m2*). The longitudinal muscle strands have diversified into multiple muscular elements and display a metameric pattern. *lmp* and *lmp-d* terminate in the sixth pleon segment, while the telson remains free of musculature (Figure 6o, -p). **(Ph V)** Close to hatching, embryos of *P. hawaiensis* display additional diversification within the existing muscle pattern (Figure 6i). The mandible is equipped with three lateral extrinsic muscles (*md-l1*, *md-l2, md-l3/mx1-l1*). The second maxilla shows three medial extrinsic muscles (*mx2-m1*, *mx2-m2*, *mx2-m3*) and two lateral extrinsic muscles (*mx2-l1*, *mx2-l2*). The telson is still devoid of musculature (Figure 6q).

### Semaphoront specification

**Figure 7:**
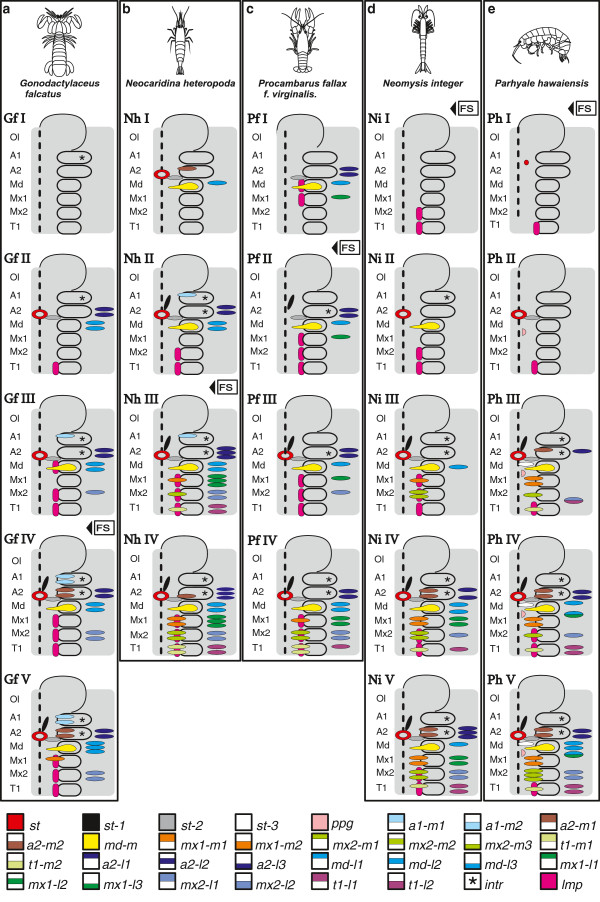
**Schematic overview of head muscle development and summary of results presented in Figures **2**-**5**. a***Gonodactylaceus falcatus*, **b***Neocaridina heteropoda*, **c***Procambarus fallax f. virginalis*, **d***Neomysis integer*, **e***Parhyale hawaiensis*. The specified semaphoronts (roman numbers) are shown as simplified drawings containing the optic lobes and anterior six hemisegments of the left body half. Muscle precursors are color coded. The color code is shown at the bottom of the figure. Lateral or medial extrinsic precursors of one segment are numbered in the order they are positioned from anterior to posterior. Abbreviations: **Gf (I-IV)**, **Nh (I-V)**, **Pf (I-IV)**, **Ni (I-V)**, **Ph (I-V)** species- and semaphoront affiliation; Ol optic lobes, A1, A2, Md, Mx1, Mx2, T1 appendage anlagen, **FS** developmental event: full set of segment anlagen present, intrinsic appendage muscle precursors are marked by asterisks.

**Figure 8:**
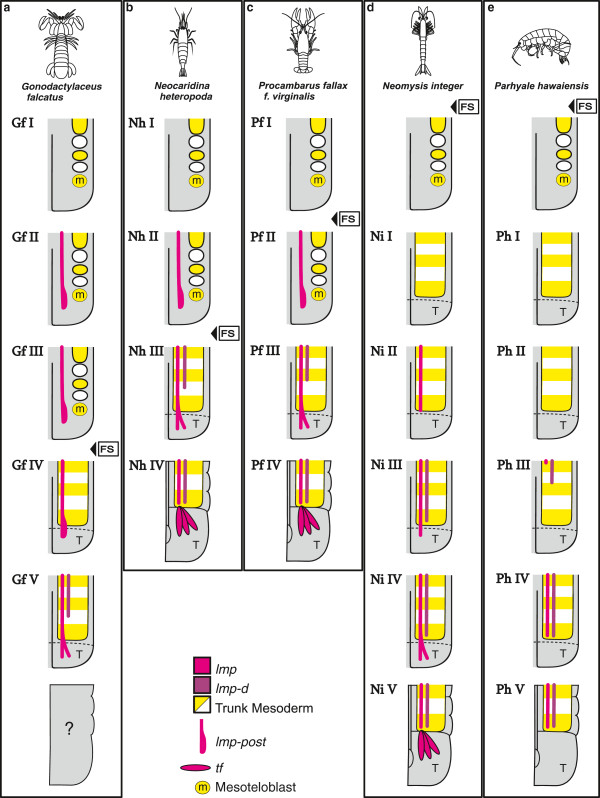
**Schematic overview of muscle development in the posterior germ band, posterior pleon- and telson anlagen.** Summary of results presented in Figure 6. **a***Gonodactylaceus falcatus*, **b***Neocaridina heteropoda*, **c***Procambarus fallax f. virginalis*, **d***Neomysis integer*, **e***Parhyale hawaiensis*. For *G. falcatus* the late embryonic stages are unknown. The specified semaphoronts (roman numbers) are presented as simplified drawings. Only hemisegments of the right body half are shown. Anterior is oriented to the top in all panels. *lmp* and *lmp-d* are shown as continuous lines and color coded (pink and purple respectively). The posterior pioneer muscle strand *lmp-post* is treated as a single unit though it cannot be precisely delineated from *lmp*. The mesoteloblasts and mesodermal cells are shown as circles with colors alternating yellow and white in a-p direction. Only one of four mesoteloblast cells is shown (yellow). Units of the trunk mesoderm, in which cell proliferation has progressed beyond the stereotypic pattern of mesodermal cell rows, are shown in the same color code. Abbreviations: **Gf (I-IV)**, **Nh (I-V)**, **Pf (I-IV)**, **Ni (I-V)**, **Ph (I-V)** (species- and semaphoront affiliation); **FS** (developmental event: full set of segment anlagen present), T telson anlage, m mesoteloblast.

Semaphoronts described in the *Results*-section are shown in a schematic overview in Figure 7 and Figure 8. 45 muscle precursors are specified (Table 1) and color coded. One color is used for *lmp-md*, *lmp-mx1*, *lmp-mx2* and *lmp-t1*. The lateral extrinsic muscle precursors of one segment are shown separately but also only assigned one color. The same is true for the medial extrinsic precursors of one segment. Five different semaphoronts could be identified for *G. falcatus*, *N. integer* and *P. hawaiensis*, while four of them could be distinguished in *N. heteropoda* and *P. fallax f. virginalis*. Schematic drawings of myogenesis in the posterior embryonic region (Figure 8) refer to the same semaphoronts also shown in Figure 7.

**Figure 9:**
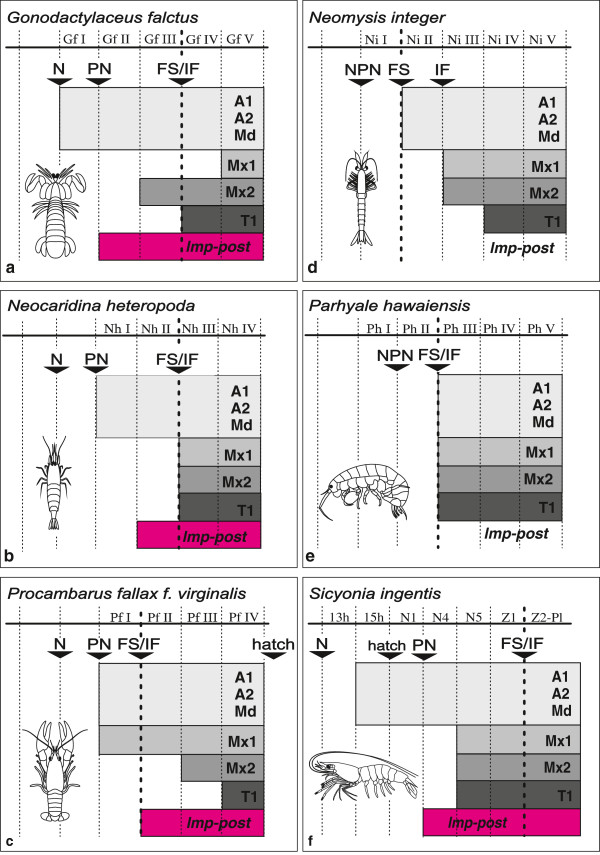
**Simplified timeline representation of developmental events. a***Gonodactylaceus falcatus*, **b***Neocaridina heteropoda*, **c***Procambarus fallax f. virginalis*, **d***Neomysis integer*, **e***Parhyale hawaiensis*, **f***Sicyonia ingentis*. Ontogenetic data on extrinsic appendage muscle development of the naupliar-, first- and second maxilla-, and first thoracopod segments, are combined to four general categories and compared between species. A more detailed comparison of myogenic sequences between species is given in Additional file 2: Figure S2. Extrinsic muscle precursors and posterior longitudinal muscle precursors in the respective semaphoronts are mapped in the sequence they first occur in each species. Categories of extrinsic muscle precursors, namely of the nauplius segments, the first maxilla segment, the second maxilla segment and the first thoracic segment, are shown in specific shades of grey. The posterior pioneer muscle strand (*lmp-post*) is also mapped (pink). General developmental events (**N**, **PN**, **FS**, **IF**) are added in the sequence they occur relative to the muscle precursors. **FS** is marked by a bold vertical dotted line. Regular vertical dotted lines mark the chronological boundaries between all semaphoronts. Abbreviations: **Gf (I-IV)**, **Nh (I-V)**, **Pf (I-IV)**, **Ni (I-V)**, **Ph (I-V)** species- and semaphoront affiliation, **N** appendage anlagen in nauplius segments present, **PN** appendage anlagen in postnaupliar segments present, **FS** full set of segment anlagen present, **IF** intersegmental furrows present in entire trunk.

Since Malacostraca exhibit a conserved number of body segments (5 head segments, 8 thorax segments, 6 pleon segments) segment position is used as overall reference for comparison of development. Four additional morphological features were used to align the temporal sequences of muscle development (Figure 9, Additional file 2: Figure S2): **N**, the ‘egg-nauplius’-stage, showing prominent appendage buds of the first antenna, second antenna and mandible; **PN**, presence of postnaupliar appendage buds (at least one appendage bud of first maxilla to sixth pleopod); **FS**, the emergence of the full set of segments, meaning that the ectodermal and mesodermal cell material responsible for generating all postnaupliar segments of the adult has been proliferated from the ectoteloblasts and mesoteloblasts respectively (the mesoteloblasts, can no longer be detected once **FS** is acquired because their directional proliferation has ceased and cell division has continued within the mesodermal units); **IF**, presence of intersegmental furrows (Here the embryo is developed to a degree where segmentation is visible externally over the entire length of the trunk and the telson is clearly delineated from the last pleomeres, as shown in Additional file 2: Figure S2). For *N. integer* and *P. hawaiensis***N** and **PN** can be taken as one event (**NPN**) (Figure 9d and -e) due to the lack of significant temporal difference between naupliar and postnaupliar appendage formation in these species. **FS** and **IF** coincide in all species investigated at the given temporal resolution, except for *N. integer*.

## Discussion

### Cephalic muscle development of Malacostraca

Comparison between investigated species reveals that stable chronological order is not prevalent in muscle development. For example the set of muscle units that appear first in development differs greatly. For *G. falcatus* it consists of just the intrinsic muscles of the first antenna (Figure 7a), in *N. heteropoda* we find stomodeal muscles (*st*, *st-2),* medial extrinsic primordia of the second antenna and mandible (*a2-m1*, *md-m*) and lateral extrinsic precursors of the mandible (*md-l1*) (Figure 7b). In *P. fallax f. virginalis* we find the lateral muscle precursor of the stomodeum (*st-2*) along with lateral extrinsic muscles of the second antenna, mandible and first maxilla (*a2-l1*, *a2-l2*, *md-1 l*, *mx1-l1*), medial extrinsic precursor of the mandible (*md-m*) and longitudinal muscle precursors in the mandible- and first maxilla segment (*lmp-md*, *lmp-mx1*) (Figure 7c). In *N. integer* myogenesis begins with the longitudinal muscle precursors which form a strand, beginning in the second maxilla segment (Figure 7d). In *P. hawaiensis* the longitudinal muscle strands beginning in the first thoracic segment (*lmp-t1*) are the major components of the initial pattern, together with muscle pioneer cells of the stomodeal muscle ring (*st*) (Figure 7e). Also subsequent patterns of cephalic muscle precursors in the ontogenetic series vary greatly.

All investigated species exhibit ventral longitudinal muscle strands (shown in pink in Figure 7) at some point in development. Metameric organization of longitudinal muscle precursors in the anterior six segments is problematic to see in some cases, especially in early stages (Figure 5e). However, metameric organization of longitudinal musculature becomes evident eventually and therefore precursors are shown as individual units (*lmp-md*, *lmp-mx1*, *lmp-mx2* and *lmp-t1*) for the respective segments. Also a dorsal longitudinal strand (*lmp-d*) is formed in the cephalic region of all species investigated. However the position of the anterior end of the dorsal strand is not clear in every case and its large distance to the appendage buds makes it difficult to assign segment positions to *lmp-d* primordia. They are therefore excluded from further discussion. In *P. fallax f. virginalis* the first ventral longitudinal precursors to appear are found in the segment of the mandible and first maxilla (*lmp-md*, *lmp-mx1*), in *N. integer* and *N. heteropoda* they are located in the segment of the second maxilla (*lmp-mx2*), but in the first thoracic segment in *G. falcatus* and *P. hawaiensis* (*lmp-t1*). Previous studies of malacostracan development gave strong indications that the first maxilla segment has a developmental origin that differs from that of the more posterior segments, which are formed by teloblastic proliferation [31,41,57,68,70]. It has been observed in several species that differentiation of the first maxilla appendage anlage is delayed, compared to the more posterior appendages. Interestingly, formation of the longitudinal muscle precursor in this segment (*lmp-mx1*) is very dynamic in our species. It is formed only after *lmp-md*, *lmp-mx2* and *lmp-t1* in *G. falcatus* and only after *lmp-mx2* in *N. heteropoda* and *N. Integer*. In *P. fallax f. virginalis* it is present already at the earliest myogenic stage together with *lmp-md*, while it is never formed in *P. hawaiensis. G. falcatus* also lacks lateral extrinsic muscles in the second maxilla segment throughout all semaphoronts investigated here. We conclude that the delay of muscle differentiation in the first maxilla segment compared to the posterior segments belongs already to the eumalacostracan ground pattern.

Despite the variation in temporal and spatial patterns of muscle development in the species investigated, *G. falcatus*, *N. heteropoda* and *P. fallax f. virginalis* show onset of myogenesis before the offset of segment proliferation (**FS**) from the mesoteloblasts and the delineation of all segment primordia by formation of intersegmental furrows (**IF**) (Figure 7a, -b and -c, Figure 9a, -b and -c, Additional file 2: Figure S2a, -b and -c). In both *N. integer* and *P. hawaiensis,* myogenesis is delayed compared to overall body patterning. This is reflected by the fact that germ band elongation is completed (**FS**) before the onset of myogenesis in these species (Figure 7d and -e, Figure 9d and -e, Additional file 2: Figure S2d and -e). In *P. hawaiensis* even full external segmentation is visible (**IF**) and the podomeres of the head- and thoracic appendages are distinguishable before muscle precursors appear. In *G. falcatus*, *N. heteropoda* and *N. integer* early onset is observed in the formation of extrinsic appendage muscles of the nauplius segments (**Gf II** in Figure 7a, Figure 9a, Additional file 2: Figure S2a, **Nh I** and **Nh II** in Figure 7b, Figure 9b, Additional file 2: Figure S2b, **Ni II** in Figure 7d, Figure 9d, Additional file 2: Figure S2d), though in *N. integer* this includes only *md-m*. In *P. fallax f. virginalis* extrinsic muscle primordia of the first maxilla, together with muscle primordia in the naupliar segments appear in advance compared to the more posterior ones (**Pf I** in Figure 7c, Figure 9c, Additional file 2: Figure S2c). In *P. hawaiensis* extrinsic muscle precursors of naupliar and postnaupliar segments appear synchronously with no temporal gap in the onset of development (**Ph II** in Figure 7e, Figure 9e, Additional file 2: Figure S2e).

### Posterior longitudinal muscle development in Malacostraca

The different ontogenetic events of posterior longitudinal muscle development are summarized schematically in Figure 8. Following the previous findings of extensor- and flexor muscle development in the pleon of *P. fallax f. virginalis*[52] we find it reasonable to assume an equivalent scaffolding role of these strands for the adult longitudinal muscle pattern of *G. falcatus* and *N. heteropoda*. The first muscle primordium detectable in the posterior region of the ventral longitudinal strand (*lmp-post*) shows strong correspondences in *G. falcatus*, *N. heteropoda* and *P. fallax f. virginalis*. This primordium was described as ‘posterior longitudinal muscle origin’ in *P. fallax f. virginalis*[52] and is formed before the full set of segments is present as mesodermal anlagen (**FS**) or visible in external morphology (**IF**) (Figure 8a, -b, and -c). It is located very close to the mesoteloblasts in the caudal papilla and also the newly formed mesodermal cells, which have been formed by them, representing the mesodermal units of the adult body segments. Also no metameric pattern, not even metameric arrangement of nuclei within the primordium is seen. These features justify the interpretation of *lmp-post* as an independent posterior longitudinal muscle primordium or ‘pioneer muscle strand’, which is not formed from mesodermal somites and hence from the mesoteloblast cells, but more likely from a separate mesodermal origin, most likely coming from the telson [68]. This interpretation is in accordance to Weygoldt [39] who observed anterior migration of telson mesoderm in embryos of the decapod *Palaemonetes varians*. The developmental consequence of a posterior pioneer muscle primordium is a continuous muscle strand connecting the telson to more anterior segments before the germ band is fully segmented (**FS, IF**) (**Gf II** in Figure 8a, **Nh II** in Figure 8b, **Pf II** in Figure 8c). The fate of *lmp-post* cannot be determined with certainty, but in *G. falcatus*, *N. heteropoda* and *P. fallax f. virginalis* we find strong support that it eventually forms the flexor muscles of the telson, because the position of *lmp-post* and *tf* show strong correspondence and no additional muscle precursors are observed in the telson at any time. Also the dorsal strand (*lmp-d*) appears to remain anterior to the telson, a feature also observed for *N. integer* and *P. hawaiensis*, and therefore is unlikely to contribute to the *tf*-muscles. Although we cannot exclude the possibility that mesodermal cells from anterior segments contribute to these muscles and that *lmp-post* merely serves as a scaffold for muscle morphogenesis which progresses by posterior migration, we find the existence of an independent posterior longitudinal muscle origin (*lmp-post*) to be much more consistent with our observations. Both peracarids, *N. integer* and *P. hawaiensis*, do not exhibit a posterior longitudinal muscle origin (*lmp-post*) as observed in *G. falcatus*, *N. heteropoda* and *P. fallax f. virginalis* (Figure 8d and -e, Figure 9d and -e, Additional file 2: Figure S2d and -e). Rather the longitudinal muscle strands (*lmp*) follow a clear anterior-posterior gradient of differentiation. The posterior-most pleon segments therefore are the last ones in which longitudinal muscle primordia are formed. In *N. integer*, which possesses a decapod-like tail fan, flexor muscles are formed in the telson nevertheless. Our observations suggest that the longitudinal strand eventually extends into the telson and gives rise to them, but without any posterior advance in differentiation (Figure 8d). Typically for an amphipod crustacean [71] and unlike *N. integer, P. hawaiensis* lacks a tail fan and possesses a comparatively small telson in the juvenile stage. Telson flexor muscles are lacking completely in *P. hawaiensis* (Figure 8e).

### The validity of the egg-nauplius concept

Correspondences between functional larvae, such as the anostracan and dendrobranchiate nauplius, and embryonic stages, such as the malacostracan egg-nauplius, are problematic, because fully differentiated, functional tissues are compared with undifferentiated, developing tissues. The free swimming nauplius larva itself is generated from specific embryonic stages, (which also correspond to the egg-nauplius) and those should also be compared. In the taxa which have no nauplius larva the egg-nauplius is followed by an advanced embryonic stage comprising additional segment anlagen and first muscle precursors. For this reason, when development between malacostracan species is compared, it is better to consider developmental trajectories, more specifically the chronological sequence of developmental events necessary to form a specific semaphoront, rather than just a single semaphoront of the developmental sequence. The developmental trajectory of nauplius-larva formation should include the events, from early embryonic anlagen to hatching, which are necessary to establish the tissue structure of the functional larva. Furthermore the developmental trajectories of nauplius-larva formation should affect all tissues of the nauplius segments which must be functional at hatching (the epidermis and developing exoskeleton, connective tissue, nervous system, digestive system, vascular system and of course musculature). The egg-nauplius concept as formulated by Scholtz [7] implies that the developmental system involved in formation of a free swimming nauplius larva is active in species which exhibit an egg-nauplius stage but lack a free-swimming nauplius larva. If, as suggested by Williams and Dahms [46,47] the egg nauplius was part of a crustacean phylotypic stage, or as argued by Scholtz [7], the nauplius larva “[…] *was conserved in the egg nauplius, which is still characterized by advanced development in the naupliar region* […],”. correspondences in the developmental trajectory should be found in more than just the epidermal tissue. In this light an egg-nauplius would be expected to include anlagen of musculature, which, however, is not the case in any of the species studied herein. The egg-nauplius stage (**N**) of *G. falcatus*, *N. heteropoda*, *P. fallax f. virginalis* and *N. integer*, is free of detectable muscle precursors. Only after postnaupliar segment formation becomes apparent in external morphology (**PN**) they are formed. To date the only published documentation comparable to our approach, which describes muscle development in a species with a free swimming nauplius larva, has been performed on a malacostracan, the dendrobranchiate decapod *Sicyonia ingentis* (Figure 9f, Additional file 2: Figure S2f) [54]. In this species nauplius muscle precursors are formed in an egg-nauplius stage, an embryo with distinct appendage buds in the nauplius segments. Muscle precursors are by no means formed synchronously in this species. Rather myogenesis begins with lateral extrinsic precursors of the second antenna (corresponding to *a2-l*), followed by the lateral extrinsic precursors of the mandible, the first antenna and the respective medial extrinsic precursors (corresponding to *a1-l*, *md-l, a1-m*, *a2-m*, *md-m*), prior to hatching (Additional file 2: Figure S2f). At nauplius stage 4 longitudinal muscle strands become visible, which extend from the second maxilla segment into the telson anlage, corresponding to *lmp-mx1*, *lmp-mx2*, *lmp-t1* and *lmp-post*. At nauplius stage 5 the first extrinsic muscle primordia of the first and second maxilla and the maxillipeds (*mx1-m*, *mx1-l*, *mx2-m*, *mx2-l*, *t1-m*, *t1-l*) appear. Kiernan and Hertzler [54] also described muscle morphology in nauplius stages of the branchiopod *Artemia salina*, although not in the preceding embryonic stages. Yet, as with *S. ingentis*, also *A. salina* must possess an embryonic phase in which larval tissues are formed through a series of developmental events. An egg-nauplius stage should therefore be present also in this species and must contain developing musculature. If we compare the egg-nauplius of *G. falcatus*, *N. heteropoda* and *P. fallax f. virginalis* with that in *S. ingentis* (and the expected egg-nauplius in *A. salina*) we see that epidermal- and muscle development are uncoupled in our species and that the egg-nauplii are not directly comparable. Nevertheless a distinct gap remains between naupliar and postnaupliar muscle formation. This finding suggests that part of an active ancestral developmental trajectory within muscle development related to nauplius larva formation is still present and can be assigned to the ground pattern of Eumalacostraca or perhaps further down the tree (as no data is available for Leptostraca). The egg-nauplius concept therefore describes a phenomenon of heterochrony in parts of the developmental system (i.e. in the epidermis), but not the whole. Our results are in accordance with Alberch et al. [72], who argues that the concept of heterochrony targets only specific traits of the organism, in this case of the embryo or larva. A theory of recapitulation (in an inclusive interpretation) of an ancestral nauplius larva must be rejected.

### An approach to the evolution of malacostracan myogenesis

**Figure 10:**
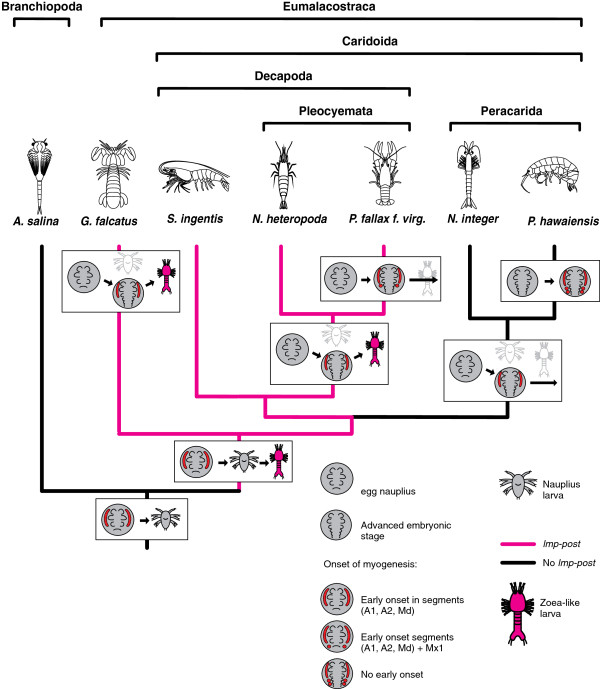
**Phylogram of hypothesized evolutionary history of myogenesis.** Phylogenetic relationships refer to [19,51]. Anostraca are used as an outgroup without implying that they are sister group to Eumalacostraca or Malacostraca. Dendrobranchiata are included, represented by *Sicyonia ingentis*, [54]. Evolutionary changes of myogenesis are shown as inferred from comparison of the simplified myogenic sequences shown in Figure 9. Only extrinsic muscle precursors and the posterior pioneer muscle strand are considered and the three nauplius segments are combined to one category. The features plotted on the tree are: egg-nauplius, advanced embryonic stage, nauplius larva, zoea-like larva, early onset of myogenesis in embryonic naupliar segments, early onset of myogenesis in embryonic naupliar segments and the first maxilla segment, lack of advanced myogenesis in nauplius segments relative to postnaupliar segments. The features are shown as small icons. Loss of either larval form is indicated by a ‘ghost’-icon. Presence of a posterior pioneer longitudinal muscle strand (*lmp-post*) is coded to the branches (pink). Absence of *lmp-post* is given in black. A free swimming nauplius larva is part of the ground pattern and has been lost three times independently in the Stomatopoda, Pleocyemata and Peracarida. In clades which possess *lmp-post*, zoea-like larvae are commonly found, indicating that both features are dependent upon each other. Loss of a zoea-like larva is clearly derived in *N. heteropoda* and *P. fallax f. virginalis*. Therefore a zoea-like larva is shown for the last common ancestor. Abbreviations: A1, A2, Md, Mx1 Body segments bearing respective appendages.

**Figure 11:**
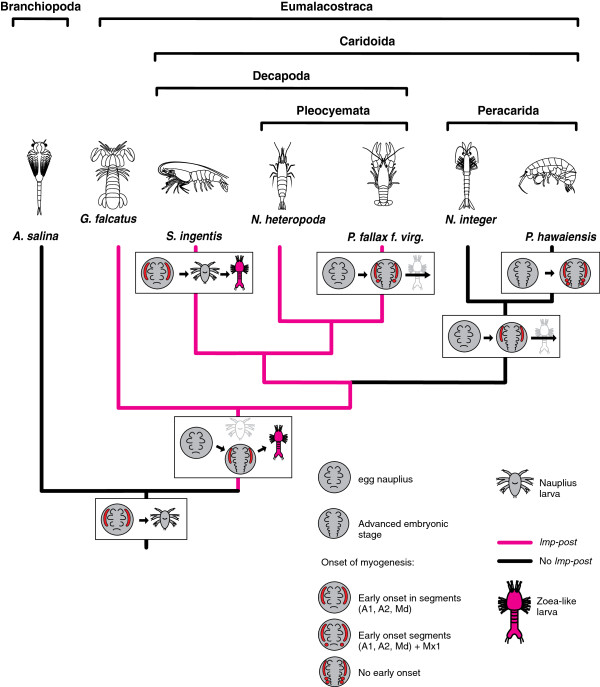
**Phylogram of hypothesized evolutionary history of myogenesis.** Phylogenetic relationships, taxa and compared developmental features as in Figure 10. An egg-nauplius stage is part of the ground pattern and the free swimming nauplius larva evolved independently in dendrobranchiates (and euphausiaceans, not shown). Abbreviations: A1, A2, Md, Mx1 Body segments bearing respective appendages.

Recent attempts to infer phylogenetic relationships from different sources of molecular data yield contradictory results [73-75]. We follow the phylogeny of Malacostraca proposed by Richter & Scholtz [19] which is based on morphological data and shown in a simplified form in Figure 10 and Figure 11. Within Eumalacostraca the stomatopods, represented by *G. falcatus* are the sister group to the remaining taxa. *N. heteropoda*, *P. fallax f. virginalis* and *S. ingentis* form the clade Decapoda. Decapoda and Peracarida appear as sister groups in the simplified tree because Euphausiacea and Anaspidacea are omitted.

The phylogenetic relationships of Malacostraca proposed by Richter & Scholtz [19] imply that Dendrobranchiata and Euphausiacea evolved from ancestors which lacked a nauplius larva. In this light the entomostracan and the malacostracan nauplius larva are not homologous. Furthermore Scholtz [7] listed several properties of the dendrobranchiate (and euphausiacean) nauplius larvae which are not shared with nauplius larvae of non-malacostracans, but with malacostracan egg-nauplii: lack of a labrum, lack of articulations in the appendages, relatively low cell number, an undifferentiated growth zone, rudimentary stomodeum and proctodeum, sparse setation, undeveloped midgut and lack of mandibular gnathobase and masticatory spines of second antenna. According to Scholtz [7] these features can be taken as additional support for the hypothesis of independent origin of the nauplius larva in dendrobranchiates (and euphausiaceans). Kiernan & Hertzler [54] describe differences in the functional muscular pattern of nauplius larvae of *S. ingentis* and *A. salina*, which are consistent with the secondary-evolution hypothesis of the dendrobranchiate nauplius. Our observations of muscle development in five malacostracan species, however, show that corresponding muscle precursors can give rise to diverse patterns of juvenile musculature. We think that homology of embryonic musculature of Branchiopoda and Malacostraca should not be excluded based on these findings alone. If the embryonic muscle precursors of *A. salina* and *S. ingentis* are homologous, an egg-nauplius stage *with* extrinsic appendage muscle precursors can be postulated for the malacostracan ground pattern. Certainly independent evolution of the dendrobranchiate and euphausiacean nauplius larvae cannot be concluded from observation of potential symplesiomorphies of egg-nauplii and nauplius larvae alone. A phylogenetic test of these developmental features based on the phylogeny of Richter & Scholtz [19], however, can be used to argue in favor of secondary nauplius larva evolution (Figure 10 and Figure 11).

If, hypothetically, a free-swimming nauplius larva was present in the malacostracan (and eumalacostracan) last common ancestor (Figure 10), this ground pattern also included an egg-nauplius stage with muscle precursors, as observed in *S. ingentis*. This pattern would then have been transformed by a heterochronic delay in embryonic nauplius muscle formation three times independently in the lineages leading to the Stomatopoda, Pleocyemata and Peracarida, which resulted in an egg-nauplius stage lacking muscle precursors. These transformations were accompanied by the loss of the nauplius larva as a hatching stage and with an advanced onset of postnaupliar segment formation and -differentiation in embryogenesis. If, however, an egg-nauplius (but no succeeding nauplius larva) was part of the eumalacostracan ground pattern (Figure 11), as part of direct development or development with an advanced larva, this egg-nauplius stage would also have lacked muscle precursors, as observed for *G. falcatus*, *N. heteropoda* and *P. fallax f. virginalis*. In this case loss of a nauplius larva and of muscle precursors in the preceding egg-nauplius stage would have occurred in the malacostracan stem lineage. The re-acquisition of a nauplius larva in Dendrobranchiata (and Euphausiacea, not shown in Figure 10 and Figure 11) would have included an acceleration of muscle development in the nauplius segments, resulting in an egg-nauplius stage with muscle precursors. This transformation would have been accompanied by an accelerated differentiation also of the non-muscular tissues of the nauplius segments, an earlier hatching event and a delay in embryonic development and differentiation of postnaupliar segments. The latter scenario (Figure 11) is clearly more parsimonious, as it requires a single loss of egg-nauplius musculature and single reacquisition in Dendrobranchiata (as well as another reacquisition in Euphausiacea, not shown). On the contrary multiple loss of the nauplius larva (Figure 10) implies three evolutionary losses of egg-nauplius musculature (not counting Leptostraca, Anaspidacea Syncarida and Thermosbaenacea, which would require four more instances of reduction), which is clearly less parsimonious.

The early onset of muscle development in the naupliar segments described for *G. falcatus* and *N. heteropoda* (Figure 9a and -b) refers only to the extrinsic appendage muscles. In either of the solutions presented above the pattern of myogenesis was altered in *P. fallax f. virginalis* so that the initial set of extrinsic appendage muscle precursors includes the naupliar segments and also the first maxilla segment (Figure 9c). The nauplioid larva of mysids, more precisely the advanced formation of first- and second antenna and the early timing of hatching are likely to be relics of a developmental trajectory including a free swimming larva. However, the early onset of appendage development is in no way reflected by the developmental pattern of extrinsic musculature. Only the medial extrinsic appendage muscle precursor (*md-m*) arises before the remaining extrinsic appendage muscle precursors (Additional file 2: Figure S2d). Since *md-m* in the mysid juvenile represents a comparatively large muscle the early onset of its development can also be related to the fact that a relatively large amount of muscle tissue has to be generated from this precursor, and not that early emergence of *md-m* is caused by cryptic larva development. Yet, given the considerably nauplius-related characteristics of the ‘nauplioid’ larva we favor an interpretation in which mysids have retained part of the ancestral myogenic program. In this light early onset of naupliar myogenesis, as proposed for the eumalacostracan last common ancestor, would have been present also in the ground pattern of Peracarida. *P. hawaiensis* shows the most derived characteristics of muscle development among the investigated species. Myogenesis is completed rapidly and certain muscle primordia observed here are not found in the other species (for example *ppg*, *md-l3/mx1-l*, *mx2-l/t1-l*). In this species the early onset of extrinsic appendage muscle development in the naupliar segments was lost (Figure 9e).

Posterior longitudinal muscle development involving *lmp-post* is interpreted as part of a developmental trajectory of myogenesis in zoea-like larval forms, because it allows the posterior pleon segments to function in movement of the trunk before differentiation of these segments is complete. In combination with a broad, paddle-shaped telson, a rapid escape movement (similar to the tail flip reflex of adult decapods [19]) could be performed by these larvae. *lmp-post* is not found in *A. salina*[54] which lacks a zoea-like larva. We conclude that *lmp-post* is an autapomorphy of Eumalacostraca (or Malacostraca, the situation in Leptostraca is unknown). Within the Malacostraca *lmp-post* is commonly a feature of embryogenesis, but is also observed in the larval phase of the dendrobranchiate *S. ingentis*. Nauplius stage 4 of *S. ingentis* is reported to exhibit developing muscle strands that extend from the second maxilla segment into the furcal processes [54]. These strands precede the longitudinal trunk musculature of the protozoea larva, which extend through the trunk into the telson before the full set of pleon segments can be distinguished externally [55]. The common occurrence of zoea-like larval forms in ontogeny of stomatopods and marine decapods leads us to conclude that *lmp-post* is a crucial element of development and – together with a zoea-like larval form- part of the eumalacostracan ground pattern (Figure 10 and Figure 11). It has been retained in these lineages, even if the zoea-like larva was lost with the adaptation to a freshwater environment (as in *N. heteropoda* and *P. fallax f. virginalis*). This conclusion is further supported by findings which suggest that zoea-like larvae can be lost and reacquired multiple times, as in the genus *Macrobrachium*[76]. In the peracarid lineage *lmp-post* was lost (Figure 10 and Figure 11) together with other embryonic relics of larva myogenesis. These reductions are most likely connected to the evolutionary acquisition of advanced brood care using a marsupium in Peracarida.

## Conclusions

The observations on muscle development in five representatives of Malacostraca contribute to our understanding of the changes in the developmental system that may have caused evolutionary transitions between larval- and embryonic phases. Also we conclude from our study that concepts of recapitulation of a larva stage, such as the egg-nauplius [46,47] are incomplete. First of all functional larval- or adult organs and tissues, must always develop from specific organ- or tissue- anlagen which, in the case of the nauplius larva, are formed already in embryogenesis. This implies that a nauplius larva and an egg-nauplius do not represent alternative situations. Rather the nauplius larva must be viewed as a hatching stage which directly follows an egg-nauplius stage. The correspondences possibly representing recapitulated ancestral features do not reside in overall morphology of embryos but in the ontogenetic trajectories of developing tissues or tissue parts. Our data shows that the sequence of emerging muscle precursors related to formation of a nauplius larva are partly retained in embryogenesis of species with advanced larval stages or direct development, but do not correspond to morphogenesis of the ectoderm. Our observations demonstrate that heterochronic events which can be discussed in the light of larva recapitulation can take place at different rates in different tissues, even the particular organ anlagen therein. Retention of larva morphology must therefore always be discussed across all stages of pre-adult ontogeny. However, in-depth investigation of malacostracan development in further tissues using cladistic methods for heterochrony analysis is beyond the scope of the present study and will be dealt with in the future.

## Competing interests

The authors hereby declare that they have no competing interests.

## Authors’ contributions

All three authors conceived the project and approved the final version of the manuscript. GJ carried out the immunohistochemistry, microscopy, computational data processing, and drafted the manuscript. SR und CW critically revised the manuscript and collected material of *G. falcatus*. All authors read and approved the final manuscript.

## Supplementary Material

Additional file 1: Figure S1 External morphology of ventral posterior germ band region shown by nuclear staining with TOPRO-3 (cyan). The first stage that reveals intersegmental furrows throughout the entire trunk is shown for embryos of *G. falcatus*, *N. heteropoda*, *P. hawaiensis* and a nauplioid larva of *N. integer*. Anlagen of pleomeres 4 to 6 are demarcated by dotted lines. Brackets point out the anteroposterior expansion of the telson anlage in **a**, **b** and **c**. In *N. integer* and *P. hawaiensis* uropod anlagen are visible at this stage. **a Gf IV**. **b Nh III**. **c Nh II**.** d Ph III**. Abbreviations: UP uropod anlagen, T Telson anlage, P4-P6 Pleon segments 4–6. Scalebars are 100 μm in all panels. Click here for file

Additional file 2: Figure S2 Detailed timeline representation of developmental events. **a***Gonodactylaceus falcatus*, **b***Neocaridina heteropoda*, **c***Procambarus fallax f. virginalis*, **d***Neomysis integer*, **e***Parhyale hawaiensis*, **f***Sicyonia ingentis*. All Muscle precursors shown in Figure 7 are considered and mapped in the sequence they occur, as well as *lmp-post*, but not *lmp-d*. The color code from Figure 7 is used. For lateral and medial extrinsic appendage muscle precursors the color specifies segment affiliation. Gross morphological features (N, PN, FS, IF) are added in the sequence they occur relative to the muscle precursors. FS is marked by a bold vertical dotted line. Abbreviations: **Gf (I-IV)**, **Nh (I-V)**, **Pf (I-IV)**, **Ni (I-V)**, **Ph (I-V)** species- and semaphoront affiliation, N appendage anlagen in nauplius segments present, PN appendage anlagen in postnaupliar segments present, FS full set of segment anlagen present, IF intersegmental furrows present in entire trunk, *Intr* intrinsic muscle precursors. Click here for file
