# Supplementary figures and images for: Myogenesis of Malacostraca – the “egg-nauplius” concept revisited
Source: Front Zool. 2013 Dec 11;10:76. doi: 10.1186/1742-9994-10-76 (PMC3903077; doi:10.1186/1742-9994-10-76)

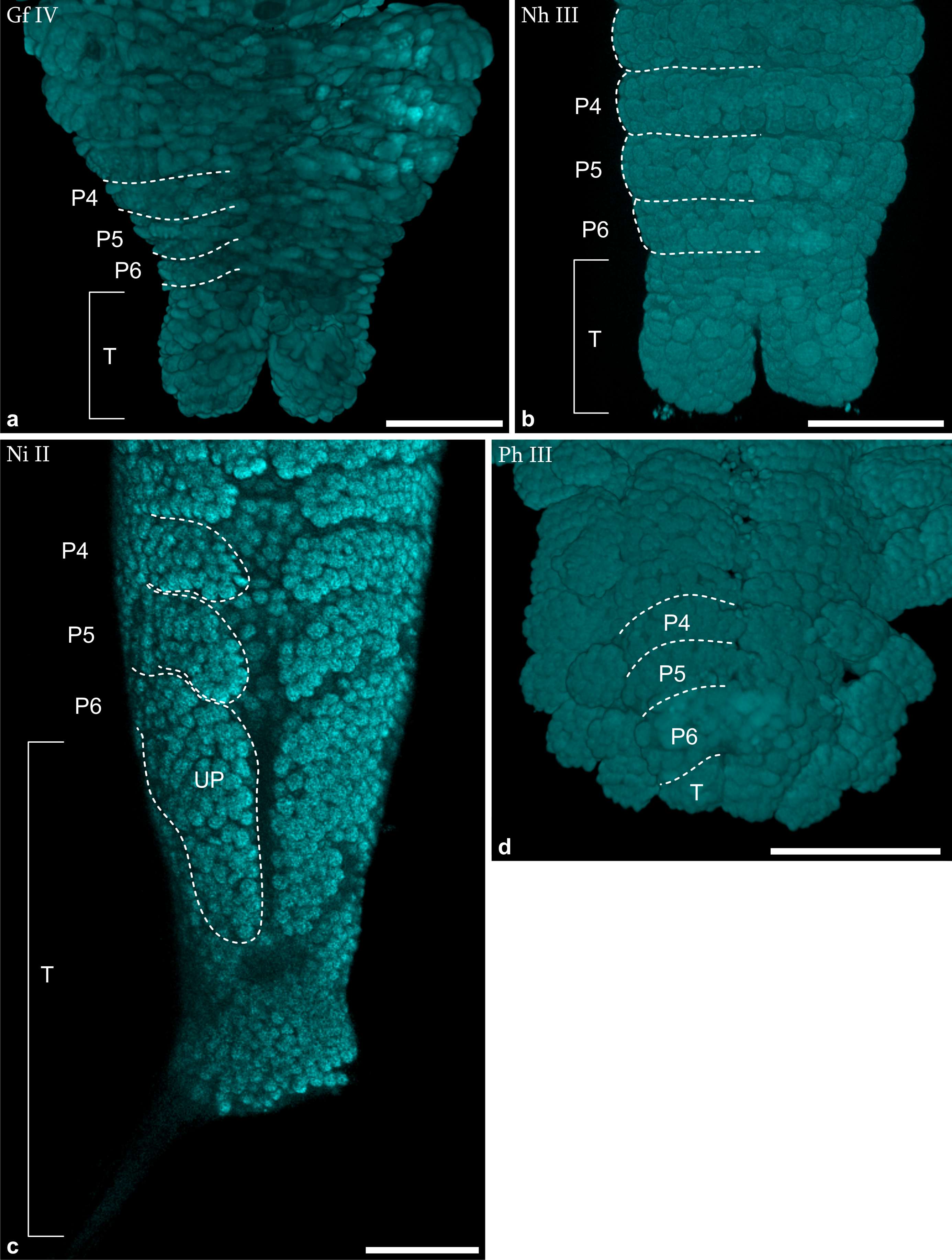

Supplement: Additional file 1: Figure S1 — External morphology of ventral posterior germ band region shown by nuclear staining with TOPRO-3 (cyan). The first stage that reveals intersegmental furrows throughout the entire trunk is shown for embryos of G. falcatus, N. heteropoda, P. hawaiensis and a nauplioid larva of N. integer. Anlagen of pleomeres 4 to 6 are demarcated by dotted lines. Brackets point out the anteroposterior expansion of the telson anlage in a, b and c. In N. integer and P. hawaiensis uropod anlagen are visible at this stage. a Gf IV. b Nh III. c Nh II. d Ph III. Abbreviations: UP uropod anlagen, T Telson anlage, P4-P6 Pleon segments 4–6. Scalebars are 100 μm in all panels. [file 1742-9994-10-76-S1.jpg]

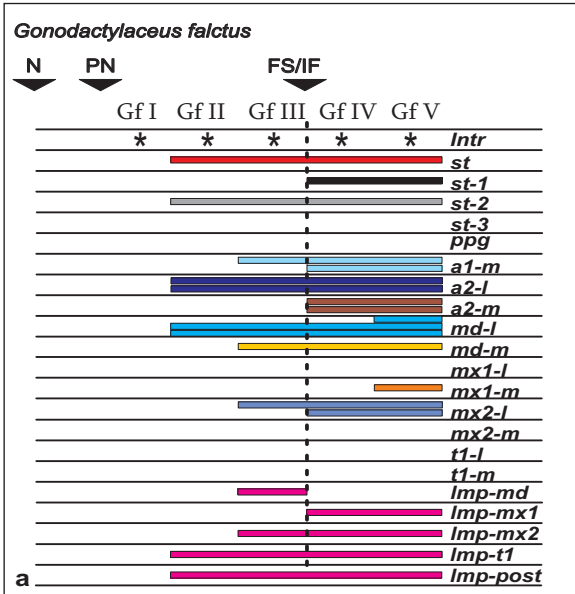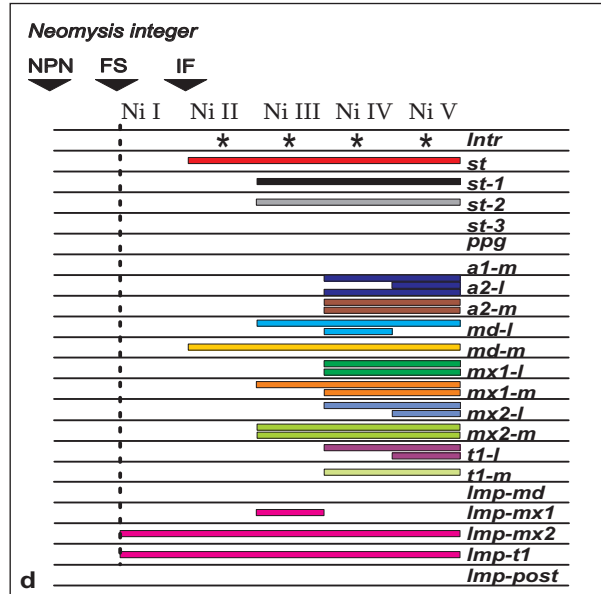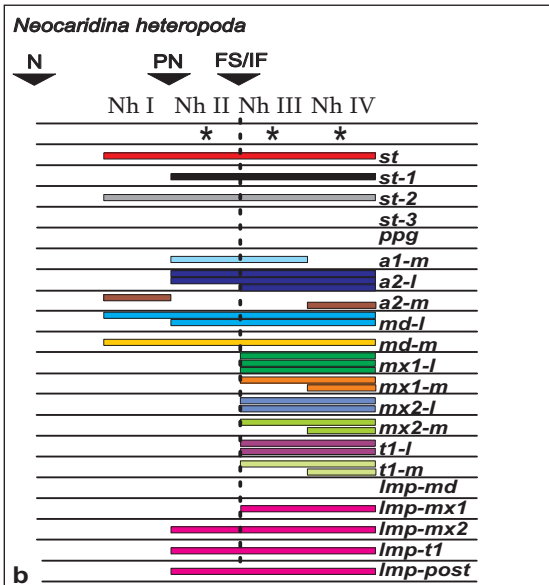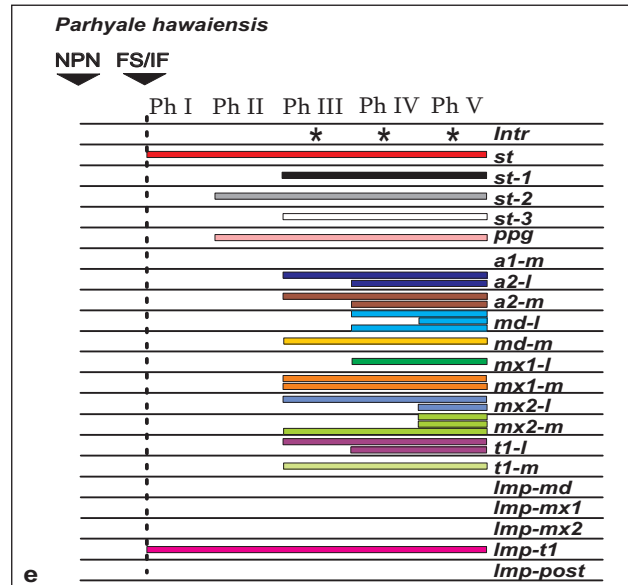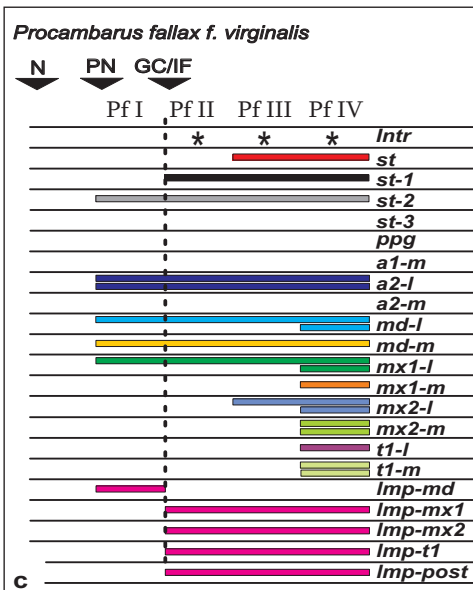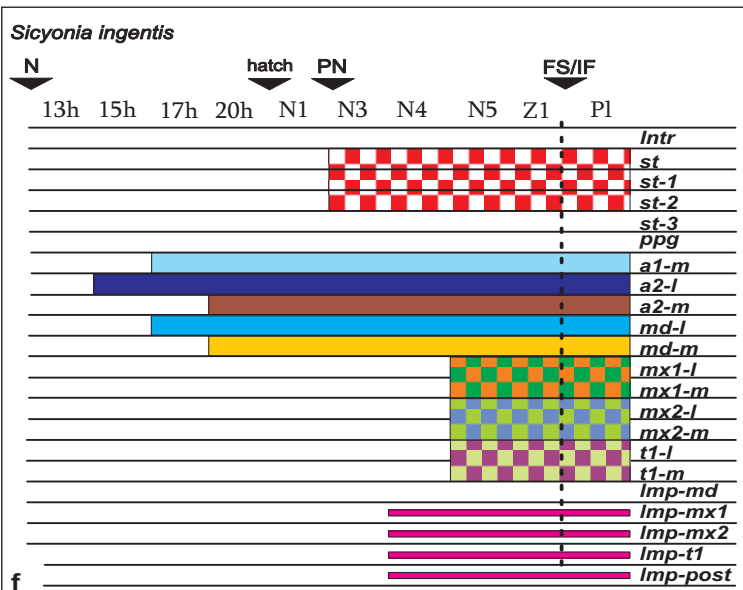

Supplement: Additional file 2: Figure S2 — Detailed timeline representation of developmental events. aGonodactylaceus falcatus, bNeocaridina heteropoda, cProcambarus fallax f. virginalis, dNeomysis integer, eParhyale hawaiensis, fSicyonia ingentis. All Muscle precursors shown in Figure 7 are considered and mapped in the sequence they occur, as well as lmp-post, but not lmp-d. The color code from Figure 7 is used. For lateral and medial extrinsic appendage muscle precursors the color specifies segment affiliation. Gross morphological features (N, PN, FS, IF) are added in the sequence they occur relative to the muscle precursors. FS is marked by a bold vertical dotted line. Abbreviations: Gf (I-IV), Nh (I-V), Pf (I-IV), Ni (I-V), Ph (I-V) species- and semaphoront affiliation, N appendage anlagen in nauplius segments present, PN appendage anlagen in postnaupliar segments present, FS full set of segment anlagen present, IF intersegmental furrows present in entire trunk, Intr intrinsic muscle precursors. [file 1742-9994-10-76-S2.pdf]
